# The role of organizational characteristics on the outcome of COVID-19 patients admitted to the ICU in Belgium

**DOI:** 10.1016/j.lanepe.2020.100019

**Published:** 2020-12-23

**Authors:** Fabio Silvio Taccone, Nina Van Goethem, Robby De Pauw, Xavier Wittebole, Koen Blot, Herman Van Oyen, Tinne Lernout, Marion Montourcy, Geert Meyfroidt, Dominique Van Beckhoven, Belgian Society of Intensive Care Medicine and the Belgian Collaborative Group on COVID-19 Hospital Surveillance

**Affiliations:** a Department of Intensive Care, Erasme Hospital, Université Libre de Bruxelles (ULB), Brussels, Belgium; b Department of Epidemiology and Public Health, Sciensano, Brussels, Belgium; c Department of Intensive Care, Cliniques Universitaires Saint-Luc, UCLouvain, Brussels, Belgium; d Department of Public Health and Primary Care, University of Gent, Gent, Belgium; e Department of Intensive Care Medicine, University of Leuven, Leuven, Belgium

**Keywords:** COVID-19, Mortality, Intensive care unit, Organisation, Surge

## Abstract

**Background:**

Several studies have investigated the predictors of in-hospital mortality for COVID-19 patients who need to be admitted to the Intensive Care Unit (ICU). However, no data on the role of organizational issues on patients’ outcome are available in this setting. The aim of this study was therefore to assess the role of surge capacity organisation on the outcome of critically ill COVID-19 patients admitted to ICUs in Belgium.

**Methods:**

We conducted a retrospective analysis of in-hospital mortality in Belgian ICU COVID-19 patients via the national surveillance database. Non-survivors at hospital discharge were compared to survivors using multivariable mixed effects logistic regression analysis. Specific analyses including only patients with invasive ventilation were performed. To assess surge capacity, data were merged with administrative information on the type of hospital, the baseline number of recognized ICU beds, the number of supplementary beds specifically created for COVID-19 ICU care and the “ICU overflow” (i.e. a time-varying ratio between the number of occupied ICU beds by confirmed and suspected COVID-19 patients divided by the number of recognized ICU beds reserved for COVID-19 patients; ICU overflow was present when this ratio is ≥ 1.0).

**Findings:**

Over a total of 13,612 hospitalised COVID-19 patients with admission and discharge forms registered in the surveillance period (March, 1 to August, 9 2020), 1903 (14.0%) required ICU admission, of whom 1747 had available outcome data. Non-survivors (*n* = 632, 36.1%) were older and had more frequently various comorbid diseases than survivors. In the multivariable analysis, ICU overflow, together with older age, presence of comorbidities, shorter delay between symptom onset and hospital admission, absence of hydroxychloroquine therapy and use of invasive mechanical ventilation and of ECMO, was independently associated with an increased in-hospital mortality. Similar results were found in in in the subgroup of invasively ventilated patients. In addition, the proportion of supplementary beds specifically created for COVID-19 ICU care to the previously existing total number of ICU beds was associated with increased in-hospital mortality among invasively ventilated patients. The model also indicated a significant between-hospital difference in in-hospital mortality, not explained by the available patients and hospital characteristics.

**Interpretation:**

Surge capacity organisation as reflected by ICU overflow or the creation of COVID-19 specific supplementary ICU beds were found to negatively impact ICU patient outcomes.

**Funding:**

No funding source was available for this study.

**Research in context**

**Evidence before this study**

Mortality among critically ill patients with COVID-19 admitted to intensive care units (ICU) varies significantly across countries. We searched PubMed, Science Citation Index, the Cochrane Injuries Group and Embase for all publications until October 15, 2020 and found several cohort studies reporting relevant clinical and biological variables that can significantly predict in-hospital mortality in this patients’ population, such as age, immunosuppression, renal injury, the use of invasive mechanical ventilation or elevated D-dimers levels. However, there are no available data on the role of organizational characteristics as risk factor associated with in-hospital mortality among ICU COVID-19 patients. As such, we considered to assess the prognostic role of several organizational characteristics using the Belgian clinical surveillance database for COVID-19 patients requiring ICU admission.

**Added value of this study**

Our cohort study included 1747 critically ill patients admitted to Belgian ICUs with complete discharge data on August 9, 2020; overall hospital mortality rate was 36.1%. In the multivariable model, ICU overflow (i.e. the ratio between the number of occupied ICU beds by confirmed and suspected COVID-19 patients divided by the number of recognized ICU beds reserved for COVID-19 patients) was independently associated with in-hospital mortality in the overall cohort. A high proportion of supplementary beds specifically created for COVID-19 ICU care was a risk factor for in-hospital mortality in patients undergoing invasive mechanical ventilation (n=999). In this subpopulation, the effect of overflow remains similar, although not statistically significant.

**Implications of all the available evidence**

This study found that ICU overflow and a high number of newly created ICU beds might significantly influence the outcome of critically ill patients with COVID-19. Management of the COVID-19 crisis should take these factors into account to organize ICU admissions.

Alt-text: Unlabelled box

## 1 Introduction

Since the rapid spread of the novel severe acute respiratory syndrome coronavirus 2 (SARS-CoV-2), critical care physicians have faced an increasing number of patients suffering from an acute hypoxemic respiratory failure associated with coronavirus disease 2019 (COVID-19) [1]. Many of these patients required the use of invasive mechanical ventilation (IMV), which was associated with a mortality rate between 40 and 65% [1,2]. Therapeutic interventions have therefore focused not only on reversing hypoxaemia and providing adequate organ support but also on potential treatments to decrease the viral load or the burden of the inflammatory response, thus limiting disease severity [3].

Understanding the outcome and the risk-factors for in-hospital mortality of COVID-19 patients admitted to the intensive care units (ICUs) remains a complex issue. In a large cohort of 1591 ICU patients in the area of Lombardy, 99% of them required respiratory support (i.e. mostly IMV) and ICU mortality was 26% [4]; however, study interpretation was flawed because 58% of patients were not yet discharged at the moment of data analysis. Another study described a cohort of 257 adult COVID-19 patients admitted to the ICUs in New York [5]; IMV and renal replacement therapy were used in 79% and 31% of patients, respectively. Overall mortality was 39%, although 94 (37%) patients were still hospitalised. A more recent study showed that among 5062 COVID-19 patients admitted to the ICU in England, 1547 (31%) deaths were reported, with significant between-centres differences [6].

From March 1 up to August 9, 2020, a total of 73,401 people have been tested positive for SARS-CoV-2 in Belgium, leading to 9746 deaths. Mortality was high among nursing home residents, who accounted for half of all reported deaths, although most of cases were suspected only on clinical symptoms. Overall in-hospital death occurred in 21% of admitted patients [7]. Considering the variable need for available ICU beds and resources for critically ill patients, some studies have focused on predictors of hospital mortality to better help clinicians to readdress the intensity of care and to better understand optimal patient management [8,9]. However, none of those evaluated whether factors related to surge capacity organisation, such as baseline number of recognized ICU beds, ICU overflow and creation of supplementary COVID-19 specific ICU beds, might also influence the outcome of these patients significantly. Indeed, this information is of utmost importance for health authorities in order to address future outbreaks and organise ICU capacity.

The aim of this study was to analyse clinical characteristics, use of resources and predictors of mortality of critically ill patients admitted to Belgian ICUs with COVID-19, with a particular focus on ICU organizational characteristics.

## 2 Methods

### 2.1 Study Design

This study was based on the national clinical surveillance of hospitalized patients with COVID-19 implemented by Sciensano, the Belgian Institute of Health, and supported by the Federal Health Ministry, in order to obtain a minimal health-related dataset for patients diagnosed with COVID-19 admitted to Belgian hospitals. Details on the surveillance program have been published [10]. The clinical surveillance program was applied to all Belgian hospitals/ICUs; nearly 72% of them contributed to the data collection [10]. Available ICU beds in Belgium before pandemics were 2000 (i.e. of those, 1200 were reserved for COVID-19 patients); a total of 800 additional ICU beds were created during the surge, with a total of 2000 ICU beds reserved for COVID-19 patients.

For this study, all patients with a SARS-CoV-2 infection admitted to one of the Belgian ICUs from March 1, 2020 up to August 9, 2020 and with registered admission and discharge forms were eligible. SARS-CoV-2 infection was confirmed by: a) a positive result of real-time reverse transcriptase-polymerase chain reaction assay of nasopharyngeal swabs or bronchoalveolar lavage or b) a rapid antigen tests (criteria included since the 3^rd^ of April) or c) chest computed tomography (CT) scan showing suggestive signs of COVID-19 infection, according to local radiological reports (criteria included since the 3^rd^ of April). Patients who were transferred to another hospital and/or with unknown status at hospital discharge were excluded from the final analysis. The hospital data collection was done by Sciensano, which is legally entitled for surveillance of infectious diseases in Belgium (Royal Decree of 21/03/2018). The COVID-19 clinical surveillance was authorized by the independent administrative authority protecting privacy and personal data, and Ethical approval was obtained from the ethical committee of Ghent University Hospital (BC-07507), which waived the informed consent because of the anonymous and retrospective data analysis.

### 2.2 Data collection

Clinical data reported in this study were collected from Belgian general hospitals through two online secured questionnaires in LimeSurvey filled in by hospital staff and directly saved on the central server of Sciensano. The first questionnaire was filled after admission, the second after hospital discharge or death, whichever came first. The recorded data includes demographics, method of diagnosis, delay from symptoms to hospital admission, clinical presentation at hospital admission, the use of specific therapies during the hospital/ICU stay (i.e. hydroxychloroquine, remdesivir, lopinavir/ritonavir, tocilizumab, macrolides, corticosteroids), the use of IMV or extra-corporeal membrane oxygenation (ECMO), some biological parameters on ICU admission (i.e. arterial partial pressure of oxygen, PaO_2_; arterial partial pressure of carbon dioxide, PaCO_2_; pH; arterial lactate; serum creatinine; total lymphocytes count; lactate dehydrogenase, LDH; C-reactive protein, CRP), the occurrence of a secondary infection of any origin, as well as ICU and hospital length of stay and in-hospital mortality.

### 2.3 Organizational characteristics

To assess organizational characteristics, the data of the clinical surveillance was merged based on the name of the hospital with administrative information on: a) the type of hospital (i.e. university hospital; general hospital with university characteristics; general hospital) b) the baseline number of recognized ICU beds; c) the creation of supplementary ICU beds specifically for COVID-19 care, expressed as the proportion of created beds among the total (created and recognized) ICU beds; d) “ICU overflow”, defined as the dynamic ratio between the number of occupied ICU beds by confirmed and suspected COVID-19 patients divided by the number of recognized ICU beds reserved for COVID-19 patients (i.e. 60% of the total number of recognized ICU beds). ICU overflow was calculated for each patient over an average of 8 days, the median length of stay in ICU (among all ICU patients including patients transferred out), starting from the date of ICU admission of the patient. ICU overflow was categorized as “present” (i.e. ratio ≥ 1.0) or “absent” (i.e. ratio < 1.0). The number of occupied ICU beds by confirmed and suspected COVID-19 patients at a given day in a given hospital was derived from the hospital Surge Capacity survey data collection [10].

### 2.4 Statistical analysis

Statistical analyses were performed using R software (R-version 3.6.0 and RStudio version 1.0.153). Descriptive statistics were computed for all study variables. A Shapiro-Wilk test was used, and histograms and normal-quantile plots were examined to verify the normality of distribution of continuous variables. Discrete variables were expressed as counts (percentage) and continuous variables as means ± SD or median [25th–75th percentiles], as appropriate. Demographics and clinical differences between ICU survivors and non-survivors were assessed using a chi-square, Fisher's exact test, Student's t-test, or Mann-Whitney U test, as appropriate. The difference in in-hospital mortality between different ranges of age for the whole ICU cohort and only considering those patients undergoing IMV was analysed using a chi-square test. Multivariable logistic regression analysis with in-hospital mortality as the dependent variable was performed including variables associated with in-hospital mortality (*p* < 0.2) on a univariate basis. We calculated individual differences for in-hospital mortality in each participating hospital by assuming hospital-specific random intercepts. Additional analyses were performed evaluating: a) only patients treated with IMV; b) only patients with available biological data on ICU admission; c) a dataset with tenfold multiple imputation for important prognostic baseline covariates. Odds ratios (OR) with 95% confidence intervals (CIs) were computed. A *p* < 0.05 was considered as statistically significant. Details for Statistical analyses are available in the Supplemental Material.

### 2.5 Role of funding source

No funding source was available for this study.

## 3 Results

### 3.1 Study population

**Fig. 1:**
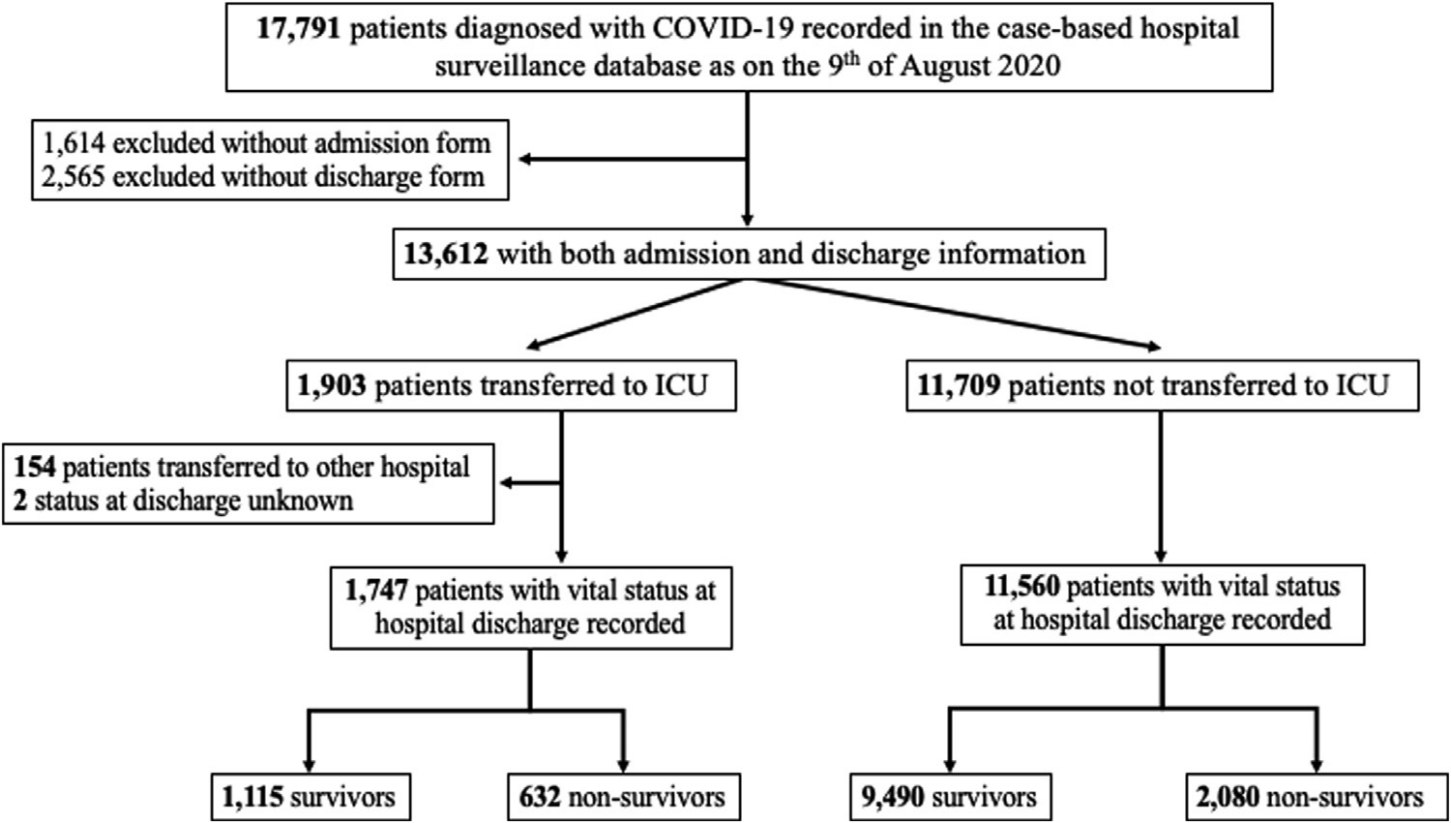
Flow-chart of the study: COVID-19 hospital clinical surveillance, Belgium, March 1^st^ – August 9^th^ 2020.

Among a total of 17,791 COVID-19 patients requiring hospital admission and recorded in the hospital clinical surveillance database during the study period, 13,612 had both admission and discharge data available. Of those, 1903 (14.0% - Fig. 1) were admitted to ICU. There were 156 patients transferred to another hospital and/or with an unknown vital status at hospital discharge, leaving a total of 1747 patients for the final analyses. There were no differences between the study population (*n* = 1747) and the overall ICU population (*n* = 1903) (Supplementary Table 1).

Median time from the onset of symptoms to hospital admission and from hospital to ICU admission were 7 [4–10] days and 1 [0–4] days, respectively. Demographic and clinical characteristics of the study population are shown in Table 1. Overall, 68.1% (1177/1728) patients were male and the median age was 66 [55–75] years. 655 (37.5%) patients were aged 71 years or older. Seventy-six percent of patients had at least one pre-existing comorbidity; the most frequent ones were arterial hypertension, cardiovascular disease and diabetes mellitus; 499 (30.6%) patients were on chronic therapy with angiotensin-converting enzyme (ACE) and/or angiotensin-II inhibitors.

**Table 1:** Characteristics of ICU-patients with available status at discharge (n=1747) according to the in-hospital mortality. COVID-19 hospital clinical surveillance, Belgium, March 1^st^ – August 9^th^, 2020. Data are presented as count (percentage) or median [IQRs].

	All patients (*n*=1747)	Survivors (n=1115)	Non-survivors (*n*=632)	*Missing*	*p value*
**DEMOGRAPHICS**					
**Age, years**	66 [55–75]	62 [51–71]	73 [64–79]	-	<0.001
* <=30, n (%)*	39 (2.2)	38 (3.4)	1 (0.1)	-	<0.001
* 31-40, n (%)*	68 (3.9)	63 (5.6)	5 (0.8)	-	<0.001
* 41-50, n (%)*	179 (10.2)	159 (14.2)	20 (3.1)	-	<0.001
* 51-60, n (%)*	354 (20.3)	264 (23.6)	90 (14.2)	-	<0.001
* 61-70, n (%)*	452 (25.9)	297 (26.6)	155 (24.5)	-	0.36
* 71-80, n (%)*	466 (26.7)	228 (20.4)	238 (37.6)	-	<0.001
* >80, n (%)*	189 (10.8)	66 (5.9)	123 (19.4)	-	<0.001
**Male Gender, n (%)**	1177 (68.1)	755 (68.1)	422 (68.1)	19	0.99
**Pre-existing comorbidities**					
**Cardiovascular Disease, n (%)**	572 (32.7)	308 (27.6)	264 (41.7)	-	<0.001
**History of arterial hypertension, n (%)**	771 (44.1)	468 (41.9)	303 (47.9)	-	0.02
**Diabetes mellitus, n (%)**	450 (25.8)	272 (24.3)	178 (28.1)	-	0.09
**Obesity (Body Mass Index > 30 Kg/m^2^), n (%)**	207 (19.2)	139 (19.3)	68 (18.8)	667	0.91
**Pre-existing pulmonary disease, n (%)**	260 (14.9)	141 (12.6)	119 (18.8)	-	<0.001
**Pre-existing neurological disease, n (%)**	97 (5.6)	63 (5.6)	34 (5.3)	-	0.89
**Cognitive disorder, n (%)**	71 (4.5)	41 (4.0)	30 (5.3)	158	0.28
**Chronic renal disease, n (%)**	190 (10.9)	79 (7.1)	111 (17.5)	-	<0.001
**Chronic liver disease, n (%)**	67 (3.8)	39 (3.4)	28 (4.4)	-	0.39
**Solid cancer, n (%)**	108 (6.2)	61 (5.4)	47 (7.4)	-	0.13
**Hematological cancer, n (%)**	39 (2.2)	21 (1.8)	18 (2.8)	103	0.25
**Presence of immunosuppression, n (%)**	48 (2.7)	24 (2.1)	24 (3.8)	-	0.06
**No comorbidities, n (%)**	415 (23.8)	316 (28.3)	99 (15.6)	-	<0.001
**Smoking, n (%)**	103 (10.5)	57 (10.1)	31 (9.9)	11	0.99
**ACEIs and/or ARBs, n (%)**	499 (30.6)	306 (27.4)	193 (30.5)	15	0.19
**Risk factors**					
**Health-care worker, n (%)**	51 (2.9)	43 (3.9)	8 (1.2)	13	0.002
**Nursing home resident, n (%)**	123 (7.1)	57 (5.1)	66 (10.4)	10	<0.001
**Disease characteristics**					
**Days from symptoms to hospital admission**	5 [2–8]	6 [3–8]	4 [2–7]	-	<0.001
**Diagnosis by RT-PCR, n (%)**	1551 (89.0)	988 (88.6)	563 (89.1)	-	0.82
**Diagnosis by chest CT-scan, n (%)**	630 (36.0)	421 (59.2)	209 (58.2)	1153	0.78
**Diagnosis by rapid antigen method, n (%)**	39 (2.0)	19 (2.7)	20 (5.6)	1103	0.02
**Body temperature on admission,**°**C**	37.6 [36.7-38.3]	37.6 [36.7-38.4]	37.6 [36.7-38.3]	-	0.31
**Weakness, n (%)**	723 (41.4)	482 (43.2)	241 (38.1)	-	0.04
**Cough, n (%)**	1032 (59.1)	697 (62.5)	335 (53.0)	-	<0.001
**Throat pain, n (%)**	111 (6.4)	86 (7.7)	25 (3.9)	-	0.002
**Rhinitis, n (%)**	73 (4.2)	60 (5.4)	13 (2.0)	-	<0.001
**Anosmia, n (%)**	61 (3.9)	50 (5.0)	11 (2.0)	201	0.004
**Dyspnea, n (%)**	1131 (64.7)	717 (64.3)	414 (65.5)	-	0.65
**Diarrhea, n (%)**	269 (15.4)	188 (16.8)	81 (12.8)	-	0.03
**Nausea/vomiting, n (%)**	181 (10.4)	131 (11.7)	50 (7.9)	-	0.01
**Headache, n (%)**	177 (10.1)	135 (12.1)	42 (6.6)	-	<0.001
**Symptoms of mental illness, n (%)**	102 (5.8)	53 (4.7)	49 (7.7)	-	0.01
**Coma, n (%)**	28 (1.6)	10 (0.9)	18 (2.8)	-	0.003
**Convulsions, n (%)**	5 (0.3)	5 (0.4)	0 (0)	-	0.99
**Pharyngitis, n (%)**	42 (2.4)	24 (2.1)	18 (2.8)	-	0.45
**Conjunctivitis, n (%)**	12 (0.7)	9 (0.9)	3 (0.4)	-	0.55
**Asymptomatic, n (%)**	43 (2.5)	34 (3.0)	9 (1.4)	-	0.04
**No clinical signs, n (%)**	126 (7.2)	83 (7.4)	43 (6.8)	-	0.69
**Abnormal pulmonary imaging, n (%)**	1618 (93.6)	1019 (92.3)	599 (95.8)	19	0.006
**Day from hospital to ICU admission**	1 [0–4]	1 [0–4]	1 [0–3]	-	0.28
**Day from symptoms to ICU admission**	7 [4–10]	8 [5–11]	7 [3–10]	-	<0.001
**PaO_2_ on ICU admission, mmHg**	68 [56–83]	69 [57–85]	66 [54–81]	364	0.01
**PaCO_2_ on ICU admission, mmHg**	36 [31–41]	36 [31–40]	37 [31–44]	363	0.004
**pH on ICU admission**	7.46 [7.40–7.49]	7.46 [7.42–7.49]	7.44 [7.35–7.48]	362	<0.001
**Lactate on ICU admission, mmol/L**	1.3 [0.9–2.0]	1.2 [0.8–1.8]	1.5 [1.1–2.5]	479	<0.001
**Creatinine on ICU admission, mg/dL**	1.03 [0.76–2.59]	0.92 [0.70–1.51]	1.38 [0.90–7.95]	537	<0.001
**Lymphocytes on ICU admission, n/mm^3^**	590 [23–1000]	740 [22–1108]	420 [14–800]	666	<0.001
**LDH on ICU admission, IU/L**	477 [355–624]	446 [334–584]	542 [405–700]	679	<0.001
**CRP on ICU admission, mg/dL**	150.4 [88–244]	128 [71–219]	176 [105-259]	513	<0.001
**Therapies and complications**					
**Mechanical ventilation, n (%)**	999 (57.1)	512 (47.1)	487 (80.5)	55	<0.001
* Age <=30, n (%)*	9 (23.1)	8 (21.0)	1 (100)		0.23
* Age 31-40, n (%)*	22 (33.8)	18 (29.5)	4 (100)		0.01
* Age 41-50, n (%)*	84 (47.5)	66 (41.7)	18 (94.7)		<0.001
* Age 51-60, n (%)*	220 (51.3)	134 (52.5)	86 (49.4)		<0.001
* Age 61-70, n (%)*	286 (64.7)	150 (51.7)	136 (89.4)		<0.001
* Age 71-80, n (%)*	296 (66.4)	121 (55)	175 (77.4)		<0.001
* Age >80, n (%)*	82 (45.6)	15 (23.1)	67 (58.2)		<0.001
**ECMO, n (%)**	63 (3.6)	21 (1.9)	42 (7.1)	108	<0.001
**Secondary Infection, n (%)**	664 (38.0)	374 (46.5)	290 (71.4)	27	<0.001
**Hydroxychloroquine, n (%)**	1308 (74.8)	859 (77.1)	449 (71.4)	7	0.01
**Lopinavir/Ritonavir, n (%)**	18 (1.0)	9 (0.9)	9 (1.4)	7	0.22
**Remdesivir, n (%)**	18 (1.0)	15 (1.3)	3 (0.4)	9	0.13
**Tocilizumab, n (%)**	36 (2.0)	25 (2.2)	11 (1.7)	11	0.59
**Macrolides, n (%)**	247 (14.1)	167 (15.2)	80 (12.7)	7	0.08
**Corticosteroids, n (%)**	332 (21.7)	204 (20.5)	128 (24.0)	198	0.13
**Outcomes**					
**ICU length of stay, days**	9 [4–19]	9 [4–20]	9 [5–18]	-	0.01
**Hospital length of stay, days**	17 [10–31]	20 [12–36]	13 [7–24]	-	<0.001
**Organizational issues**					
**General Hospital, n (%)**	1086 (62.4)	690 (62.1)	396 (62.8)	6	0.81
**General Hospital with University Characteristics, n (%)**	381 (21.8)	224 (20.1)	157 (24.9)	6	0.02
**University Hospital, n (%)**	273 (15.6)	196 (17.7)	77 (12.2)	6	0.003
**Number of recognized ICU beds**	22 [13–36]	22 [13–36]	22 [14–39]	8	0.84
**Proportion between created and total ICU beds**	0.38 [0.30–0.48]	0.38 [0.29–0.49]	0.38 [0.30–0.47]	8	0.27
**ICU overflow, n (%)**	745 (46.1)	460 (44.1)	285 (49.7)	88	0.03

ICU = intensive care unit; ECMO = extracorporeal membrane oxygenation; CRRT = continuous renal replacement therapy; ACEIs = angiotensin converting enzyme inhibitors; ARBs = angiotensin II receptor blockers;

* = with low molecular weight heparins on ICU admission

The most frequent symptoms on admission were fever, dyspnoea and cough; PaO_2_ on ICU admission was 68 [56–83] mmHg, PaCO_2_ 36 [31–41] mmHg and pH 7.46 [7.40–7.49]. Other biological variables are reported in Table 1. Invasive mechanical ventilation was used in 999/1692 (59.0%) patients; the proportion of patients treated with IMV was significantly higher in patients between 51 and 70 years of age when compared to others (Supplemental Figure 1; *p* < 0.001). ECMO was implemented in 63/1671 (3.8%) patients. Secondary infections were diagnosed in 664/1313 (50.6%) patients. Most of patients were treated with hydroxychloroquine (1308/1742, 75.1%), while a few of them received other therapies (Table 1). ICU and hospital length of stay were 9 [4–19] and 17 [10–31] days, respectively; ICU length of stay was significantly longer in patients on IMV (16 [8–26] days) and on ECMO (20 [10–35] days) than in others (4 [2–7] days; *p* < 0.001).

### 3.2 Characteristics of survivors and non-survivors

Overall mortality was 36.1%; the median time from hospital admission to death was 13 [7–24] days. In univariate analysis, non-survivors were older and had more frequently previous cardiovascular disease, a history of arterial hypertension, pre-existing pulmonary and chronic renal diseases than survivors (Table 1). In-hospital mortality progressively increased by age in all ICU patients and in patients treated with IMV (Supplemental Figure 2). There were fewer healthcare workers and more nursing home residents among non-survivors than survivors. Time from the onset of symptoms to hospital admission, but not from hospitalisation to ICU admission, was shorter in non-survivors than survivors. On ICU admission, non-survivors more frequently presented with mental confusion or coma compared to survivors. All available biological variables were significantly different between non-survivors and survivors on ICU admission. Non-survivors were also more frequently treated with IMV and ECMO, more frequently developed secondary infections and less frequently received hydroxychloroquine than survivors.

### 3.3 Predictors of in-hospital mortality

**Fig. 2:**
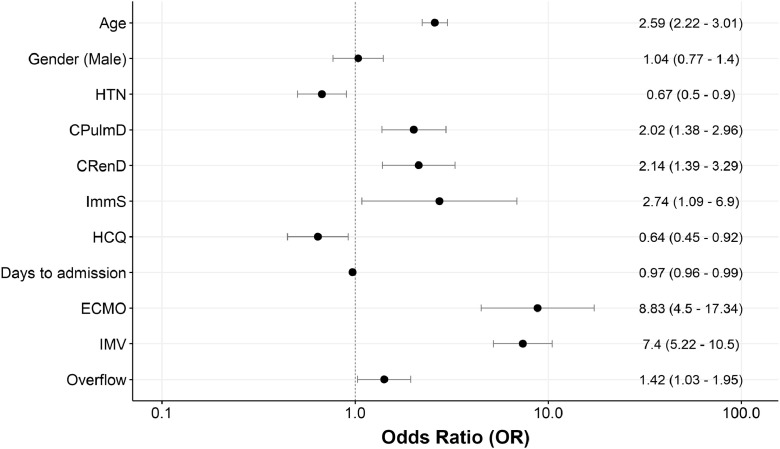
Multivariable mixed-effects model for predictors of in-hospital mortality (fixed effects) among COVID-19 patients admitted to ICU. COVID-19 hospital clinical surveillance, Belgium, March 1st – August 9th 2020. The following fixed effects were retained in the final model: age, gender, chronic immunosuppression, chronic renal disease, chronic pulmonary disease, arterial hypertension, days from symptoms to hospital admission, extra-corporeal membrane oxygenation, invasive mechanical ventilation, and overflow. Hospital was added as a random effect to the model. Odds ratio per 10 years of age is shown. Abbrevations: HCQ = hydroxychloroquine; ECMO = extra-corporeal membrane oxygenation; IMV = invasive mechanical ventilation; ImmS = chronic immunosuppression; CRenD = chronic renal disease; CPulmD = chronic pulmonary disease; HTN = arterial hypertension.

In the multivariable mixed effects analysis, older age (OR 2.59 [95% CIs 2.22–3.01] per 10 years increase), the presence of chronic pulmonary disease (OR 2.02 [1.38–2.96]), chronic renal disease (OR 2.14 [1.39–3.29]) or chronic immunosuppression (OR 2.74 [1.09–6.90]) and the absence of history of arterial hypertension (OR 0.67 [0.50–0.90]) were independently associated with in-hospital mortality (Fig. 2). A shorter delay between symptom onset and hospital admission (0.97 [0.96–0.99]), IMV (7.40 [5.22–10.50]) and ECMO (8.83 [4.50–17.34]) were also predictors of in-hospital mortality. Treatment with hydroxychloroquine was associated with a lower in-hospital mortality (0.64 [0.45-0.92]).

**Fig. 3:**
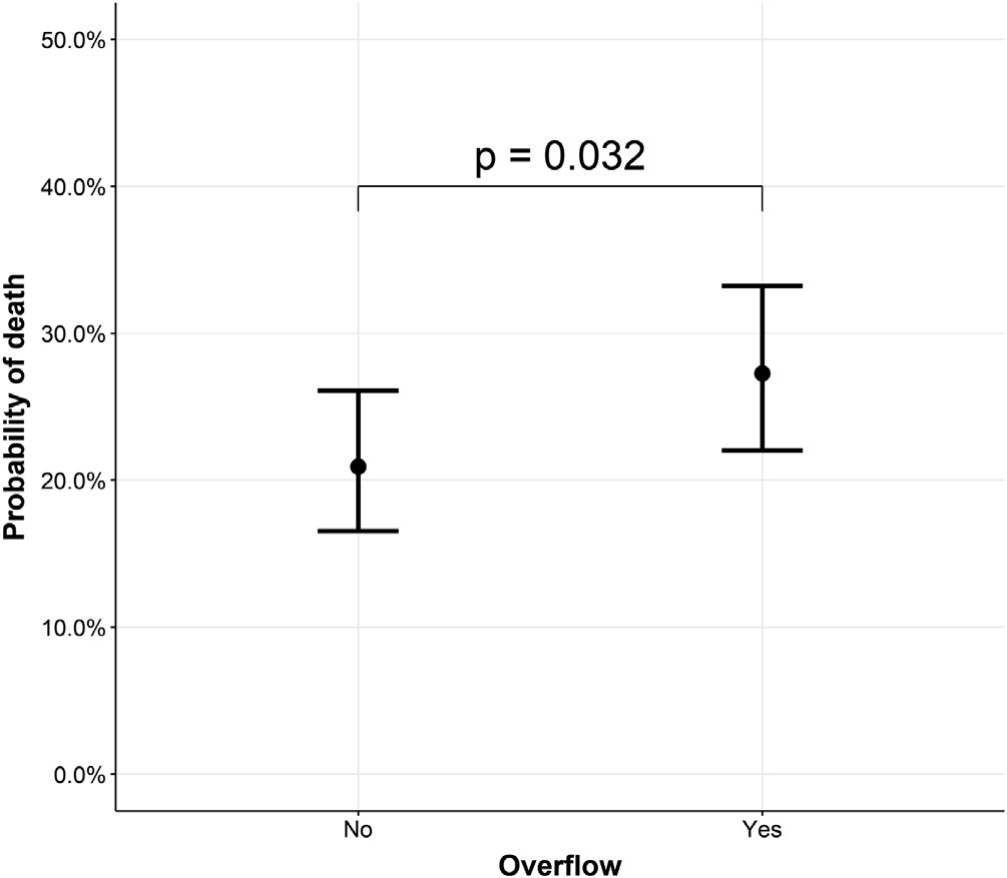
Adjusted predicted values of mortality for overflow. COVID-19 hospital clinical surveillance, Belgium, March 1st – August 9th 2020. The marginal effect of overflow is based on a mixed effects model with a random effect for each hospital and fixed effects for age, gender, chronic immunosuppression, chronic renal disease, chronic pulmonary disease, arterial hypertension, days from symptoms to hospital admission, hydroxychloroquine, extra-corporeal membrane oxygenation, invasive mechanical ventilation, and overflow. Means are used to fix continuous variables and proportions are used to fix categorical variables.

**Fig. 4:**
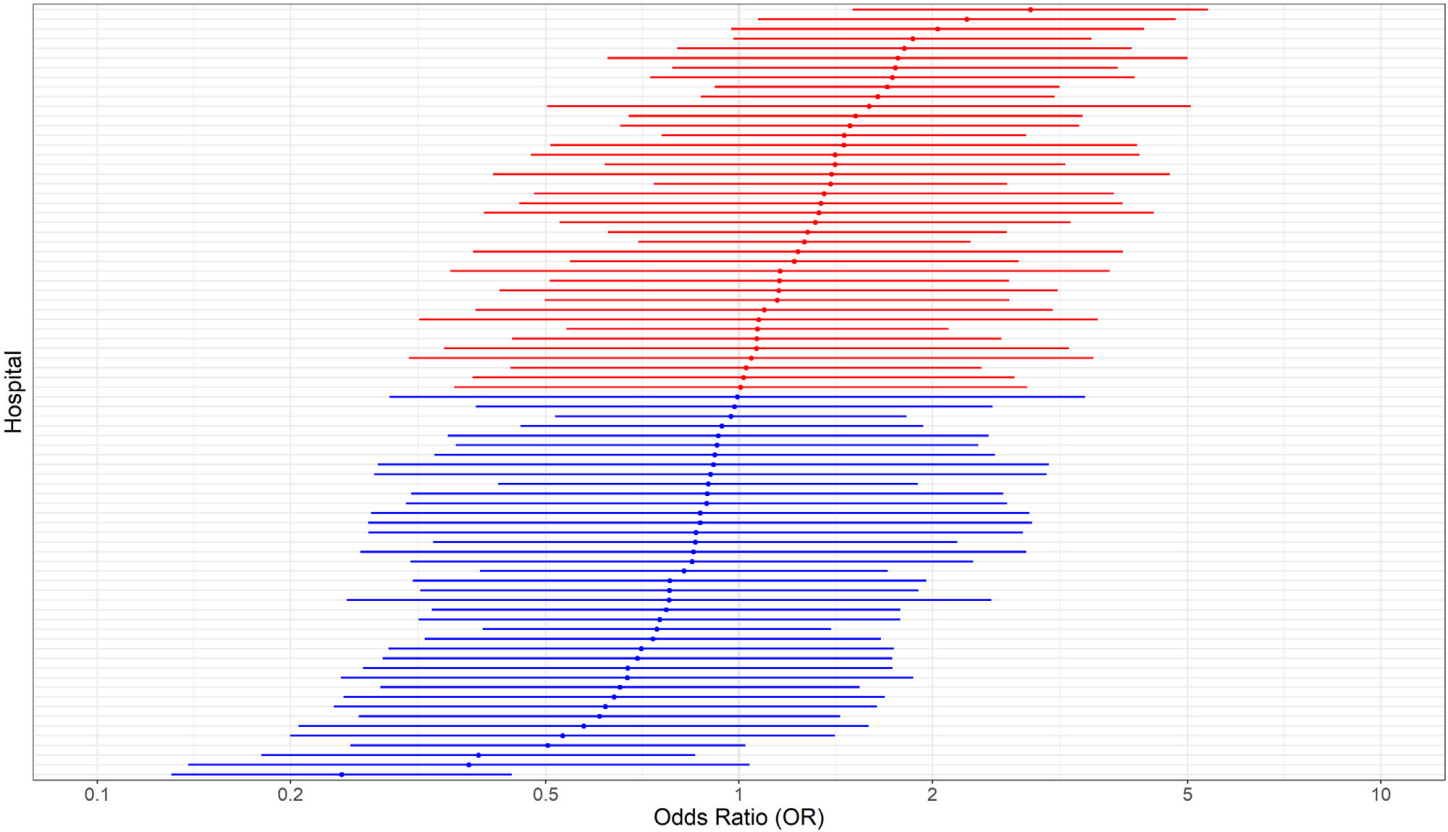
Between hospital variation in in-hospital mortality among COVID-19 patients admitted to ICU, based on a mixed effects model with a random effect for each hospital and fixed effects for age, gender, chronic immunosuppression, chronic renal disease, chronic pulmonary disease, arterial hypertension, days from symptoms to hospital admission, hydroxychloroquine, extra-corporeal membrane oxygenation, invasive mechanical ventilation, and overflow. COVID-19 hospital clinical surveillance, Belgium, March 1st – August 9th 2020. In red, hospitals with higher adjusted in-hospital mortality; in blue, hospitals with lower adjusted in-hospital mortality compared to the average over all hospitals (i.e. the global estimate for the intercept).

Although there was a significantly lower in-hospital mortality for patients admitted to university hospital (77/273, 28.2%) when compared to the others (general: 396/1086, 36.4% - general with university characteristics: 157/381, 41.2% - *p* = 0.003), this effect disappeared when accounting for the clustering effect within hospitals. When taking into account all other covariates, the number of recognized ICU beds remained negatively associated with in-hospital mortality (Supplemental Figure 3; *p* = 0.03), while the ratio between newly created ICU beds on the total number of ICU beds was positively associated with in-hospital mortality (Supplemental Figure 3; *p* = 0.002). Nevertheless, the significant association disappeared for both variables when taking into account the individual hospital as a random effect in the model (Supplemental Figure 3). The only organizational characteristic, which was independently associated with in-hospital mortality, was ICU overflow (OR 1.42 [1.03–1.95]). The adjusted probability of in-hospital mortality was 21% in the absence of ICU overflow, while it rises to 27% when ICU overflow is present, holding all other variables constant (Fig. 3). There was a significant variation in hospital mortality of COVID-19 patients admitted to ICU among different hospitals, when taking into account potential confounding variables (Fig. 4; *p* < 0.001). In the multivariable model (Supplemental Figure 4) including all patients but using imputation for missing values, the same predictors of in-hospital mortality were observed.

### 3.4 Predictors of in-hospital mortality in patients treated with mechanical ventilation

**Fig. 5:**
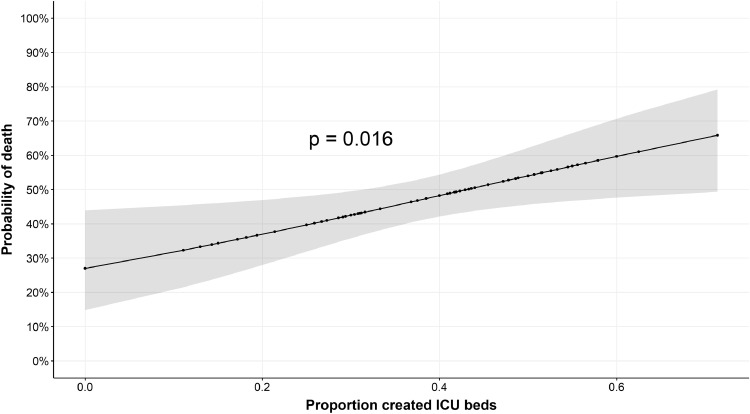
Adjusted predicted values of mortality for the proportion of created ICU beds in ventilated patients. COVID-19 hospital clinical surveillance, Belgium, March 1st – August 9th 2020. The marginal effect of the proportion of created ICU beds is based on a mixed effects model with a random effect for each hospital and fixed effects for age, gender, chronic immunosuppression, chronic renal disease, chronic pulmonary disease, arterial hypertension, days from symptoms to hospital admission, hydroxychloroquine, macrolides, extra-corporeal membrane oxygenation, invasive mechanical ventilation, nursing home resident, and the proportion of created ICU beds. Means are used to fix continuous variables and proportions are used to fix categorical variables.

In the multivariable model (Supplemental Figure 5; *n* = 999; overall in-hospital mortality 48.7% - Supplemental Table 2), older age, chronic lung disease, chronic renal disease, and chronic immunosuppression were all associated with in-hospital mortality. Being a nursing home resident was also significantly associated with mortality. Both hydroxychloroquine and macrolide therapies were associated with a significantly lower in-hospital mortality. The proportion of supplementary ICU beds specifically created for COVID-19 ICU care among the total number of ICU beds was an independent risk factor for in-hospital mortality in the mixed model, thus accounting for individual hospital differences. The adjusted predicted probabilities of in-hospital mortality according the proportion of created ICU beds are presented in Fig. 5. Non-survivors were more frequently hospitalised in hospitals with ICU overflow compared to survivors (225/445, 50.5% vs. 203/486, 41.7%; OR 1.43 [1.10–1.84] - *p* = 0.008), although no longer statistically significant in the multivariable analysis (1.39 [0.94–2.01]; *p* = 0.07).

### 3.5 Predictors of in-hospital mortality in patients with available biological data on admission

In the multivariable model (Supplemental Figure 6; *n* = 823 in total; 757 with available outcome data), older age, absence of history of arterial hypertension, chronic renal disease, a shorter delay between symptoms onset and hospital admission, the use of IMV or ECMO as well as creatinine and CRP values on admission were independent significant predictors of in-hospital mortality. Also, ICU overflow was independently associated with in-hospital mortality. Significant differences between the entire cohort and the subgroup of patients with available biological data on ICU admission are shown in Supplemental Table 3.

## 4 Discussion

In this study, we observed that hospital mortality in severe COVID-19 patients requiring admission into Belgian ICUs was 36%. A median of 38% of supplementary ICU beds specifically created for COVID-19 ICU care above the total available beds was available in Belgian ICUs during the COVID-19 pandemic. ICU organizational characteristics, such as ICU overflow (all cohort) and a high proportion of additionally created ICU beds (patients on IMV) were independently associated with in-hospital mortality, together with older age, comorbid diseases, a shorter time from the onset of symptoms to hospital admission and the severity of respiratory impairment, which was reflected by the use of IMV and ECMO. This study is the first suggesting that mortality of critically ill COVID-19 patients could be influenced by organizational factors that different health care systems had to face during this first phase of the pandemic: the rapid creation of additional beds and the challenges of local overflow, sometimes exceeding trained available ICU staffing and resource capacity.

The severity of cases and the rapidly overwhelming caseload during the COVID-19 pandemic spread has made ICUs worldwide suffer from physical, material and emotional exhaustion [11]. The high number of expected hypoxemic patients requiring ICU admission was an unseen organisational challenge, related to the availability of ICU beds and ventilators to manage the peak surge. In most countries, huge efforts had to be made to make sufficient ICU beds available by reducing or cancelling scheduled or non-urgent admissions, but also to turn hospital wards, recovery rooms, operating theatres and emergency rooms into novel ICUs [12]. A major bottleneck to this operational health care changes was the availability of adequately trained ICU personnel. This led to physical exhaustion and a high incidence of severe psychological burnout amongst ICU doctors, nurses, and physiotherapists [13].

Concomitantly, to avoid rationing of critical care services, physicians and nurses from other departments had to be deployed in the ICU, often in a so-called “tiered staffing model”, which integrated experienced ICU personnel with reassigned hospital staff members (i.e. from operating rooms, internal medicine or other wards). Although this model was the only feasible solution in this urgent situation, no data on the impact on the quality of critical care provided to COVID-19 patients, the occurrence of avoidable errors (i.e. drug preparations, weaning protocols, response to critical alarms), the adequate supervision to the tiered staff and the sufficient training of non-ICU personnel have been reported. Our results suggest that the COVID-19 pandemic has revealed the vulnerability of the organisation of the ICU healthcare system and that readdressing critically ill patients to other specialized ICUs (i.e. in the same country or towards closer international centres) might be more beneficial for patients than creating new ICU beds or taking care of a very high number of critically ill COVID-19 patients, that exceeds the usual ICU flow outside the pandemic. Collaboration with other hospitals within networks or healthcare organizations (i.e. government, transport organizations) can be helpful to ensure allocation of patients in ICUs with adequate supplies, materials and available trained personnel [12]. Importantly, lack of specific data on relevant ICU or hospital characteristics (i.e. number of ICU physicians and specialized nurses) and of valuable studies from Belgium analyzing inter-hospital variability in mortality or other outcomes limits the validity on this hypothesis to explain our observation.

Whether these data might also explain the potential relationship between higher in-hospital mortality and a lower number of available ICU beds per 100,000 population (i.e. more ICU overflow and higher number of newly created ICU beds) observed across different European countries needs to be further explored. In this setting, Belgium has one of the highest number of ICU beds per capita in Europe, with 16 beds per 100,000 inhabitants [14]. Within the subgroup analysis of patients that underwent IMV, the proportion of newly created ICU beds compared to previously existing ICU beds was an independent risk factor for mortality. This variable is likely a proxy for ICU staff qualification as smaller, less experienced centres had to create more beds to adapt to the pandemic surge. The ICU overflow variable became borderline non-significant in this subgroup likely due to decreased cohort size and statistical power.

In this study, clinical predictors of in-hospital mortality were similar to those shown in other reports. Mortality for COVID-19 patients who received IMV often exceeded 50% in several series [15,16]. Whether the use of mechanical ventilation is just the marker of lung impairment severity due to viral spread or is a determinant of poor outcome, remains poorly evaluated. In COVID-19 patients, the goal of using IMV is to save lives from severe and refractory hypoxemia, gaining time to allow lung healing. Unfortunately, data about the use of non-invasive mechanical ventilation or on respiratory function and related therapies (i.e. ventilatory parameters or modes, static compliance or driving pressure,), which can also impact patient outcomes, were lacking in this database for further adjustments [8,[17], [18], [19]]. Although a recent study reported a 60-day mortality around 35% in selected patients undergoing early ECMO support [20], in our study hospital mortality associated with the use of ECMO was 66%; whether this result is related to patients’ selection, the lack of recognized ECMO centres in Belgium or the development of ECMO-related complications, is impossible to analyse from the available retrospective data. Ongoing analyses of large registries would further clarify the role of ECMO support in COVID-19 patients. Several other studies have reported older age and the presence of several comorbid diseases as independent predictors of poor outcome [8,18,21]. Interestingly, another study also reported that a shorter median time from disease onset to ICU admission, in particular when it was shorter than one week, was associated with a higher in-hospital mortality [22]; these findings are probably related to a more rapid and severe progression of the disease, as suggested by a higher number of concomitant failing organs and had higher IL-6 levels. Although arterial hypertension was more common among non-survivors in the univariate analysis, it was found as a protective factor for mortality in the multivariate analysis. This could be because it was a proxy for patients that were not in shock as these patients may less likely be classified as having hypertension.

Another important finding is the between-centres variability in hospital mortality; this has been already reported in ICUs from United Kingdom [6], while the cause of such findings remains unclear and may have a variety of explanations, including local practices, ICU staff expertise, use of resources and unmeasured differences in patients’ cohorts. Such results may however influence the optimal decisional process to admit severe COVID-19 patients, who could be transferred to more experienced and effective ICUs, or eventually promote telemedicine to share protocols and practices among different centres.

Lastly, very few studies have shown effectiveness of therapeutic interventions in severe COVID-19 patients. Some therapies, such as remdesivir or tocilizumab, which might have some effects on the recovery of viral symptoms or reduced inflammation in adults hospitalized COVID-19 patients [23,24]. were rarely used in Belgium. Only the use of corticosteroids has been shown to effectively reduce mortality in COVID-19 patients, in particular for patients receiving oxygen therapy or treated with invasive mechanical ventilation [25]. In our study, mortality of ICU patients on steroids was 38% and the proportion of patients treated with steroids was similar between survivors and non-survivors. However, corticosteroid use for critically ill patients with COVID-19 was only recommended in Belgium after the results of randomized trials were published (i.e. mid-June 2020). Moreover, administration of these drugs could have been decided in a later phase of the disease in some patients, data on the type and daily dosages were not available, and some of these patients might have been categorized as receiving corticosteroids when they were receiving intravenous hydrocortisone for persistent hypotension requiring vasopressors; this would be associated with a higher risk of death and attenuate its impact in the statistical model. Treatment with hydroxychloroquine was associated with a lower risk of in-hospital mortality, in line with a previous analysis of the same observational Belgian surveillance data focusing on all hospitalized COVID-19 patients [26]. Despite encouraging results on the reduction of viral load and overall mortality observed in retrospective analyses [27], recent randomized trials, with inherently higher methodological quality, reported no significant effects on COVID-19 patient outcomes and a recent systematic review suggested as significant increase in mortality when combination of hydroxychloroquine and azithromycin was used [28,29]. Our data should be therefore interpreted with caution. Considering the context of previously published randomized trials and the observational nature of this study, our results likely reflect that less severely ill patients receive hydroxychloroquine.

This study has several limitations to acknowledge. First, the quality of reporting to a newly COVID-19 national hospital registration system can be challenged. Second, data collection was not developed to obtain specific ICU data (i.e. severity scores; organ failure; use of vasopressors or dialysis); some of these factors are significant determinants of patients’ outcome [6]. Also, there was some missing values, although the sensitivity analysis with imputation confirmed the main results of the study. Third, it was impossible to compare patients admitted to "recognized ICU beds" to those admitted to "newly created ICU based", as this information was missing. Moreover the "newly created ICU based" could have been created in different areas of the hospital (i.e. ward, operating room, previously unavailable ICU beds). Also, no information on the type of available “life-support therapies” (i.e. renal replacement therapy or ECMO) were available; however, all ICU beds would have a ventilator and ECMO use is available only in dedicated centers, as in other European countries. We also missed information about the nurse to patients ratio, which is 2:1 to 3:1 in Belgian ICUs; it remains therefore unknown whether this ratio has significantly changed, as local nursing teams might have been integrated with non-ICU nurses, shifts might have been changed according to local policies (i.e. 8 vs. 12 hours) and the exact number of available nurses could be different on a daily basis. Similarly, the ICU physician to patients ratio could have been different among ICUs and, as the nurse to patient ratio, influence overall outcome. Importantly, no specific information on triage for admission to recognized versus newly created ICU beds was available into the registry. Forth, we could not assess the causes of death, such as directly from COVID-19 infection, secondary complications, other events unrelated to the initial infection or withdrawal of life sustaining treatment. Fifth, we evaluated outcome during hospitalization (i.e. discharged alive vs. dead); prolonged follow-up period (i.e. up to 90 days after admission) could have provided a more reliable assessment of long-term outcome in these patients. Finally, admission criteria to the ICU may differ from other countries, which would limit the generalizability of our findings.

## 5 Conclusions

In this national dataset with an hospital mortality of 36.1% for COVID-19 patients requiring ICU admission, mortality varied significantly across different centres and was associated with organizational characteristics, in particular ICU overflow and the proportion of additionally created ICU beds. Management of crisis surges during the following waves of COVID-19 and possible other pandemics should take into account these factors to readdress patients into ICUs with the adequate quality of care and available materials and trained personnel.

## Authors' contributions

FST, NVG and DVB conceived the study; NVG, RDP and KB selected the population; NVG, MM, RDP and KB reviewed all available data; NVG, RDP, KB, HVO, TL and MM conducted the statistical analysis; FST, GM, NVG and DVB and wrote the first draft of the paper; XW, KB, HVO, TL and MM revised the text for intellectual content. All the authors have full access to the data of the present study, approved the final version of this manuscript and accepted responsibility to submit for publication. NVG, GM and DVB has verified the data of the study.

## Declaration of Competing Interests

FST received lecture fees from BD, Zoll, Nihon Khoden and Neuroptics, which are all outside the content of the present study. Other authors declare that they have no competing interests.

## Appendix Supplementary materials

Image, application 1

Image, application 2

Image, application 3

## Footnotes

Supplementary material associated with this article can be found, in the online version, at doi:10.1016/j.lanepe.2020.100019.
