# Supplementary material for: The role of organizational characteristics on the outcome of COVID-19 patients admitted to the ICU in Belgium
Source: Lancet Reg Health Eur. 2020 Dec 23;2:100019. doi: 10.1016/j.lanepe.2020.100019 (PMC7757349; doi:10.1016/j.lanepe.2020.100019)
Supplement: Supplementary file 1 [file mmc1.docx]

**Supplemental Material**

**The role of organizational characteristics on the outcome of COVID-19 patients admitted to the ICU**

*Fabio Silvio TACCONE^1*^, Nina VAN GOETHEM^2*^,Robby DE PAUW^2^, Xavier WITTEBOLE^3^, Koen BLOT^2^, Herman VAN OYEN^2,4^, Tinne LERNOUT^2^, Marion MONTOURCY^2^, Geert MEYFROIDT^5^, ^#^ Dominique VAN BECKHOVEN^2#^ on behalf of the Belgian Society of Intensive Care Medicine and the collaborative group on COVID-19 Hospital surveillance*

*^1^Department of Intensive Care*

*Erasme Hospital, Université Libre de Bruxelles (ULB),*

*Brussels, Belgium*

*^2^* *Department of Epidemiology and Public Health*

*Sciensano, Brussels, Belgium*

*^3^Department of Intensive Care*

*Cliniques Universitaires Saint-Luc, UCLouvain*

*Brussels, Belgium*

*^4^Department of Public Health and Primary Care*

*University of Gent*

*Gent, Belgium*

*^5^Department of Intensive Care Medicine*

*University of Leuven*

*Leuven, Belgium*

**equally contributed as first author*

*^#^equally contributed as senior author*

*Correspondence: Pr.* ***Fabio Silvio TACCONE***

*Department of Intensive Care*

*Hôpital Erasme*

*Route de Lennik, 808*

*1070 Brussels, Belgium*

*tel: +3225555587*

*fax: +3225554698*

*email: ftaccone@ulb.ac.be*

**Supplemental Methods**

*Data Collection*

Clinical data reported in this study were collected from Belgian general hospitals through two online secured questionnaires in LimeSurvey filled in by hospital staff and directly saved on the central server of Sciensano. The first questionnaire was filled after admission, the second after hospital discharge or death, whichever came first. The recorded data includes demographics, method of diagnosis, delay from symptoms to hospital admission, clinical presentation at hospital admission, the use of specific therapies during the hospital/ICU stay (i.e. hydroxychloroquine, remdesivir, lopinavir/ritonavir, tocilizumab, macrolides, corticosteroids), the use of IMV or extra-corporeal membrane oxygenation (ECMO), some biological parameters on ICU admission (i.e. arterial partial pressure of oxygen, PaO_2_; arterial partial pressure of carbon dioxide, PaCO_2_; pH; arterial lactate; serum creatinine; total lymphocytes count; lactate dehydrogenase, LDH; C-reactive protein, CRP), the occurrence of a secondary infection of any origin, as well as ICU and hospital length of stay and in-hospital mortality.

*Statistical Analysis*

Statistical analyses were performed using R software (R-version 3.6.0 and RStudio version 1.0.153). Descriptive statistics were computed for all study variables. A Shapiro-Wilk test was used, and histograms and normal-quantile plots were examined to verify the normality of distribution of continuous variables. Discrete variables were expressed as counts (percentage) and continuous variables as means ± SD or median [25th–75th percentiles], as appropriate. Demographics and clinical differences between ICU survivors and non-survivors were assessed using a chi-square, Fisher’s exact test, Student’s t-test, or Mann-Whitney U test, as appropriate. The difference in in-hospital mortality between different ranges of age for the whole ICU cohort and only considering those patients undergoing IMV was analysed using a chi-square test. Multivariable logistic regression analysis with in-hospital mortality as the dependent variable was performed including variables associated with in-hospital mortality (p<0.2) on a univariate basis. Potential multi-collinearity of the selected covariates was assessed based on the variance inflation factor (VIF). A mixed model with a random intercept for each participating hospital was used to model the within-hospital correlation among patients for mortality. The risk factors for in-hospital mortality were identified through a top-down model selection process that starts by fitting a full model including the variables that were chosen *a priori*. The full model and the reduced nested model are compared using the Akaike Information Criterion (AIC). The most parsimonious model was selected using this backward selection approach. We calculated individual differences for in-hospital mortality in each participating hospital by assuming hospital-specific random intercepts. The departure from the overall model estimate for intercepts was plotted. Only complete observations were included in the final analysis. Additional analyses were performed evaluating: a) only patients treated with IMV; b) only patients with available biological data on ICU admission; c) a dataset with tenfold multiple imputation for important prognostic baseline covariates; missing data among model-selected covariates were assumed to be missing at random, i.e. independent of the underlying missing values given the observed data. The AMELIA package Version 1.7.6 (Multiple Imputation of Incomplete Multivariate Data) was used to run the bootstrapping-based algorithm on the missing data and to create ten imputed datasets. The merTools package Version 0.5.2 (Tools for Analyzing Mixed Effect Regression Models) was subsequently used to extract averaged fixed effects parameters from the mixed-effect model objects fitted on the imputed datasets. The Rubin correction for combining estimates and standard errors was applied to adjust for the within and between imputation variances. Selection bias for the subset of patients with available biological data on ICU admission was evaluated by comparing baseline characteristics of cases with available biological data to the complete population included in the current study. Also, baseline characteristics between the population with a known vital status at discharge and the overall ICU population were compared. Odds ratios (OR) with 95% confidence intervals (CIs) were computed. A p < 0.05 was considered as statistically significant.

**Supplemental Results**

*Study population*

Median time from the onset of symptoms to hospital admission and from hospital to ICU admission were 7 [4-10] days and 1 [0-4] days, respectively. Demographic and clinical characteristics of the study population are shown in Table 1. Overall, 68.1% (1177/1728) patients were male and the median age was 66 [55-75] years. 655 (37.5%) patients were aged 71 years or older. Seventy-six percent of patients had at least one pre-existing comorbidity; the most frequent ones were arterial hypertension, cardiovascular disease and diabetes mellitus; 499 (30.6%) patients were on chronic therapy with angiotensin-converting enzyme (ACE) and/or angiotensin-II inhibitors.

The most frequent symptoms on admission were fever, dyspnoea and cough; PaO_2_ on ICU admission was 68 [56-83] mmHg, PaCO_2_ 36 [31-41] mmHg and pH 7.46 [7.40-7.49]. Other biological variables are reported in Table 1. Invasive mechanical ventilation was used in 999/1692 (59.0%) patients; the proportion of patients treated with IMV was significantly higher in patients between 51 and 70 years of age when compared to others (eFigure 1; p<0.001). ECMO was implemented in 63/1671 (3.8%) patients. Secondary infections were diagnosed in 664/1313 (50.6%) patients. Most of patients were treated with hydroxychloroquine (1308/1742, 75.1%), while a few of them received other therapies (Table 1). ICU and hospital length of stay were 9 [4-19] and 17 [10-31] days, respectively; ICU length of stay was significantly longer in patients on IMV (16 [8-26] days) and on ECMO (20 [10-35] days) than in others (4 [2-7] days; p<0.001).

*Additional Analyses*

In the multivariable model (eFigure 5) including only patients treated with IMV (n=999; overall in-hospital mortality 48.7% - eTable 2), older age, chronic lung disease, chronic renal disease, and chronic immunosuppression were all associated with in-hospital mortality. Being a nursing home resident was also significantly associated with mortality. Both hydroxychloroquine and macrolide therapies were associated with a significantly lower in-hospital mortality. The proportion of supplementary ICU beds specifically created for COVID-19 ICU care among the total number of ICU beds was an independent risk factor for in-hospital mortality in the mixed model, thus accounting for individual hospital differences. The adjusted predicted probabilities of in-hospital mortality according the proportion of created ICU beds are presented in Figure 3B. Non-survivors were more frequently hospitalised in hospitals with ICU overflow compared to survivors (225/445, 50.5% vs. 203/486, 41.7%; OR 1.43 [1.10-1.84] - p=0.008), although no longer statistically significant in the multivariable analysis (1.39 [0.94-2.01]; p=0.07).

In the multivariable model (eFigure 6) including only patients with available biological data upon ICU admission (n=823 in total; 757 with available outcome data), older age, absence of history of arterial hypertension, chronic renal disease, a shorter delay between symptoms onset and hospital admission, the use of IMV or ECMO as well as creatinine and CRP values on admission were independent significant predictors of in-hospital mortality. Also, ICU overflow was independently associated with in-hospital mortality. Significant differences between the entire cohort and the subgroup of patients with available biological data on ICU admission are shown in eTable 3.

**Supplemental eFigure 1:** Proportion of COVID-19 ICU patients treated with invasive mechanical ventilation by age. COVID-19 hospital clinical surveillance, Belgium, March 1^st^ – August 9^th^ 2020. N = number of patients.

**
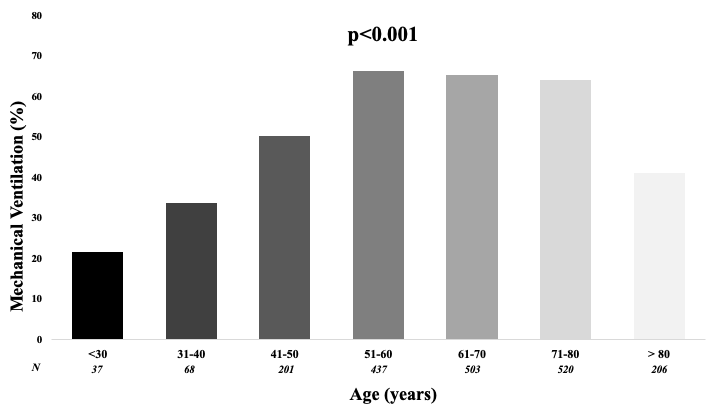
**

**Supplemental eFigure 2:** In-hospital mortality by age for all COVID-19 ICU patients (bleu histograms) and those treated with invasive mechanical ventilation (IMV; orange histograms). COVID-19 hospital clinical surveillance, Belgium, March 1^st^ – August 9^th^ 2020.


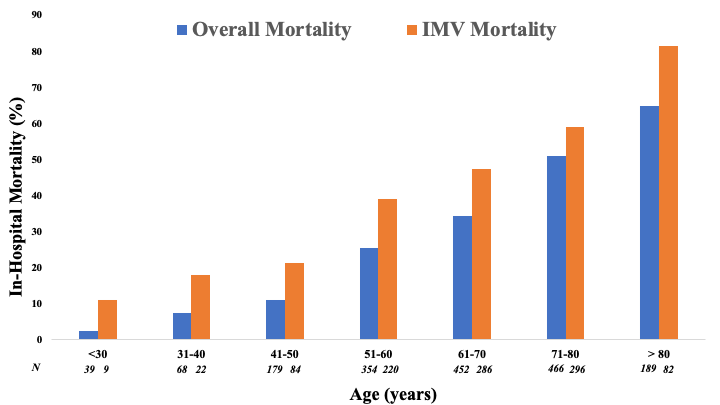


**Supplemental eFigure 3**: Adjusted predicted values of mortality for the number of recognized ICU beds (A and B) and for the proportion of created ICU beds (C and D). COVID-19 hospital clinical surveillance, Belgium, March 1st – August 9th 2020. The marginal effect is based on a fixed effects model with fixed effects for age, gender, chronic immunosuppression, chronic renal disease, chronic pulmonary disease, arterial hypertension, days from symptoms to hospital admission, hydroxychloroquine, extra-corporeal membrane oxygenation, and invasive mechanical ventilation for panels A and C. The marginal effect is based on a mixed effects model with a random effect for each hospital and fixed effects for age, gender, chronic immunosuppression, chronic renal disease, chronic pulmonary disease, arterial hypertension, days from symptoms to hospital admission, hydroxychloroquine, extra-corporeal membrane oxygenation, and invasive mechanical ventilation for panels B and D. Means are used to fix continuous variables and proportions are used to fix categorical variables.


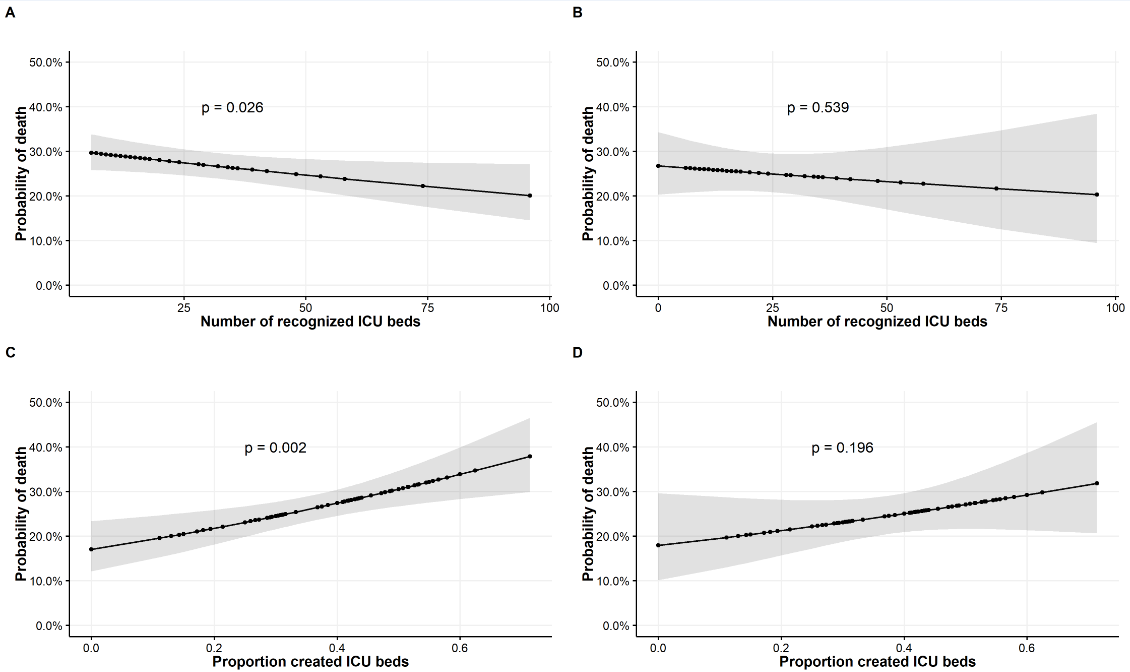


**Supplemental eFigure 4:** Multivariable model for predictors of in-hospital mortality, including all patients but using imputation for missing values. COVID-19 hospital clinical surveillance, Belgium, March 1st – August 9th 2020. Data are presented as odds ratio (OR) and 95% confidence intervals (CIs). Odds ratio per 10 years of age is shown.


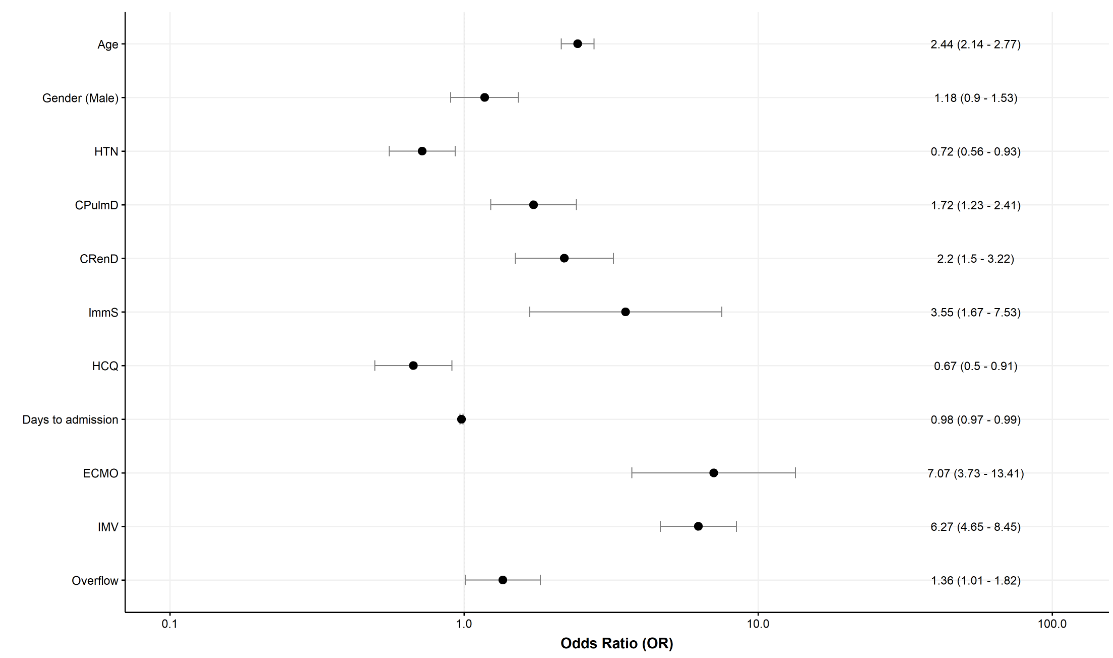


HCQ = hydroxychloroquine; HTN = arterial hypertension; ECMO = extra-corporeal membrane oxygenation; ImmS = immunosuppression; CRenD = chronic renal disease; CPulmD = chronic pulmonary disease; IMV = invasive mechanical ventilation

**Supplemental eFigure 5:** Multivariable model for predictors of in-hospital mortality, including only patients on invasive mechanical ventilation. COVID-19 hospital clinical surveillance, Belgium, March 1st – August 9th 2020. Data are presented as odds ratio (OR) and 95% confidence intervals (CIs). Odds ratio per 10 years of age is shown.


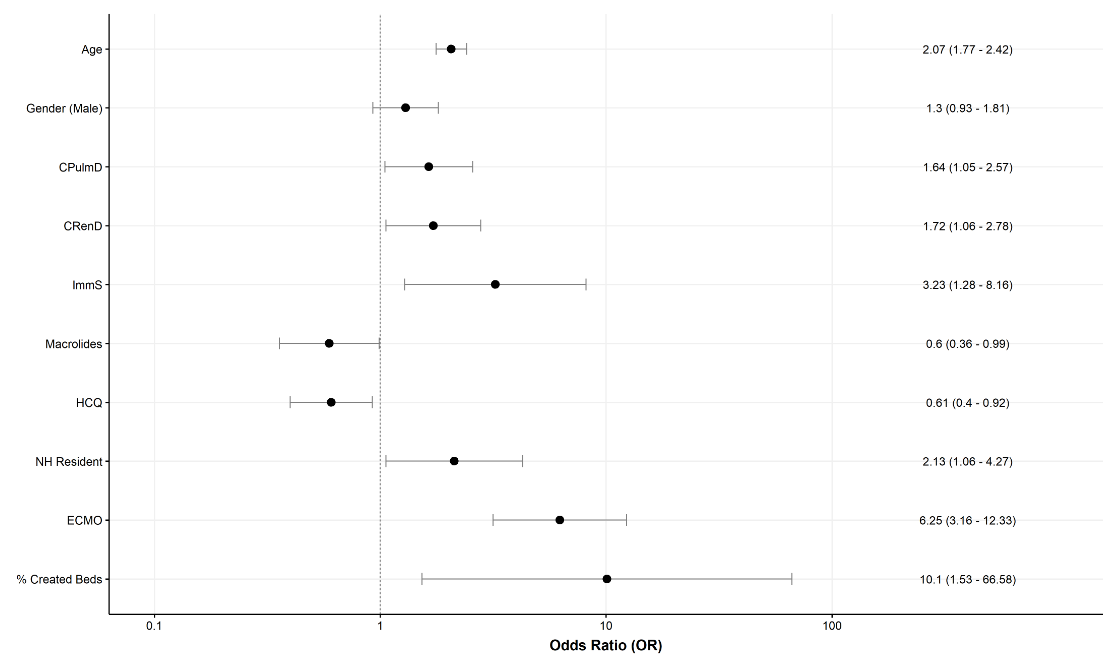


HCQ = hydroxychloroquine; ECMO = extra-corporeal membrane oxygenation; NH = nursing home; ImmS = immunosuppression; CRenD = chronic renal disease; CPulmD = chronic pulmonary disease

**Supplemental eFigure 6:** Multivariable model for predictors of in-hospital mortality, including only patients with available biological data on ICU admission. COVID-19 hospital clinical surveillance, Belgium, March 1^st^ – August 9^th^ 2020. Data are presented as odds ratio (OR) and 95% confidence intervals (CIs). Odds ratio per 10 years of age is shown.


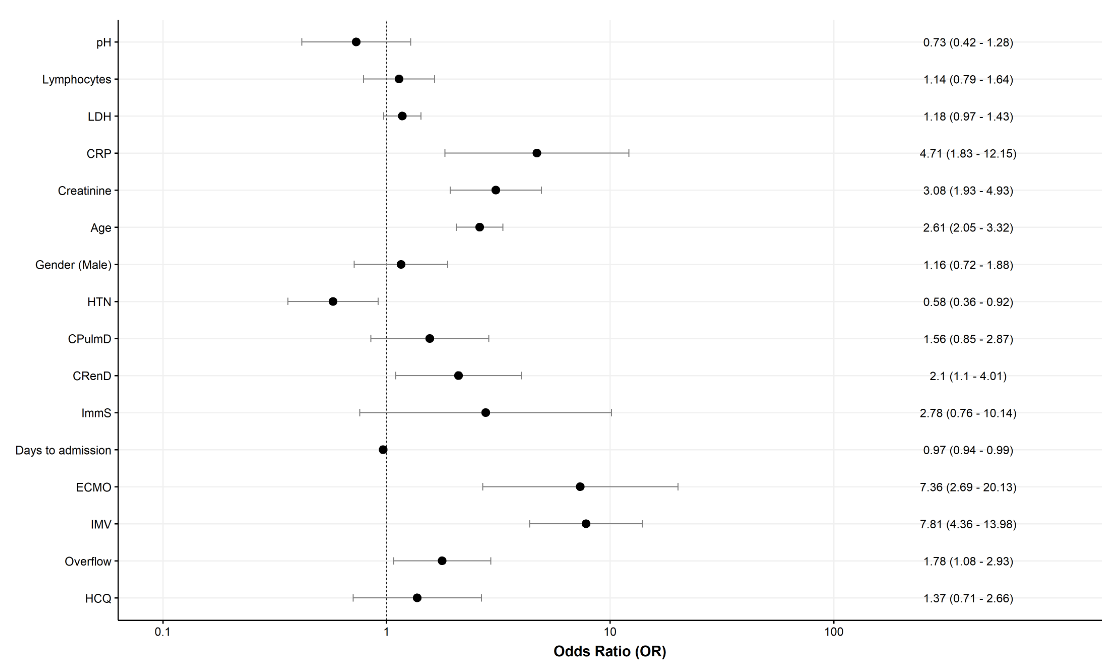


ECMO = extra-corporeal membrane oxygenation; IMV = invasive mechanical ventilation; CRenD= chronic renal disease; CRP = C-reactive protein ; HTN = arterial hypertension; HCQ = hydroxychloroquine; ImmS = immunosuppression; CRenD = chronic renal disease; CPulmD = chronic pulmonary disease; IMV = invasive mechanical ventilation

**Supplemental eTable 1.** Comparison of main characteristics of all ICU patients admitted for COVID-19 (n=1903) and those with available outcome data (n=1747). COVID-19 hospital clinical surveillance, Belgium, March 1^st^ – August 9^th^ 2020. Data are presented as median [IQRs] or count (%).

|  | **All ICU Patients (n=1903)** | **ICU Patients with available outcome (n=1747)** | ***p value*** |
| --- | --- | --- | --- |
| **Age, years** | 65 [55-75] | 66 [55-75] | 0.28 |
| **Male Gender, n (%)** | 1287 (68.3) | 1177 (68.1) | 0.91 |
|  |  |  |  |
| **Cardiovascular Disease, n (%)** | 602 (31.6) | 572 (32.7) | 0.49 |
| **History of arterial hypertension, n (%)** | 831 (43.6) | 771 (44.1) | 0.80 |
| **Diabetes mellitus, n (%)** | 484 (25.4) | 450 (25.8) | 0.85 |
| **Obesity (Body Mass Index > 30 Kg/m^2^), n (%)** | 225 (19.3) | 207 (19.2) | 0.96 |
| **Pre-existing pulmonary disease, n (%)** | 283 (14.8) | 260 (14.9) | 0.99 |
| **Pre-existing neurological disease, n (%)** | 102 (5.3) | 97 (5.6) | 0.85 |
| **Cognitive disorder, n (%)** | 72 (4.1) | 71 (4.5) | 0.72 |
| **Chronic renal disease, n (%)** | 198 (10.4) | 190 (10.9) | 0.68 |
| **Chronic liver disease, n (%)** | 68 (3.5) | 67 (3.8) | 0.74 |
| **Solid cancer, n (%)** | 113 (5.9) | 108 (6.2) | 0.81 |
| **Hematological cancer, n (%)** | 40 (2.1) | 39 (2.2) | 0.88 |
| **Presence of immunosuppression, n (%)** | 52 (2.7) | 48 (2.7) | 0.99 |
| **No comorbidities, n (%)** | 452 (23.7) | 415 (23.8) | 0.99 |
| **Smoking, n (%)** | 110 (10.5) | 103 (10.5) | 0.99 |
| **ACEIs and/or ARBs, n (%)** | 531 (27.9) | 499 (30.6) | 0.69 |
|  |  |  |  |
| **Health-care worker, n (%)** | 64 (3.3) | 51 (3.0) | 0.49 |
| **Nursing home resident, n (%)** | 124 (6.5) | 123 (7.0) | 0.56 |
|  |  |  |  |
| **Days from symptoms to hospital admission** | 6 [3-8] | 5 [2-8] | 0.41 |
| **Diagnosis by RT-PCR, n (%)** | 1696 (89.1) | 1551 (89.0) | 0.78 |
| **Diagnosis by chest CT-scan, n (%)** | 671 (58.2) | 630 (36.0) | 0.76 |
| **Diagnosis by rapid antigen method, n (%)** | 42 (3.6) | 39 (2.0) | 0.99 |
| **Day from hospital to ICU admission** | 1 [0-4] | 1 [0-4] | 0.89 |
| **Day from symptoms to ICU admission** | 7 [4-11] | 7 [4-10] | 0.51 |
| **PaO_2_ on ICU admission, mmHg** | 68 [56-83] | 68 [56-83] | 0.84 |
| **PaCO_2_ on ICU admission, mmHg** | 36 [31-41] | 36 [31-41] | 0.97 |
| **pH on ICU admission** | 7.46 [7.40-7.49] | 7.46 [7.40-7.49] | 0.78 |
| **Lactate on ICU admission, mmol/L** | 1.3 [0.9-1.9] | 1.3 [0.9-2.0] | 0.71 |
| **Creatinine on ICU admission, mg/dL** | 1.05 [0.76-3.56] | 1.03 [0.76-2.59] | 0.48 |
| **Lymphocytes on ICU admission, n/mm^3^** | 590 [24-1000] | 590 [23-1000] | 0.32 |
| **LDH on ICU admission, IU/L** | 484 [356-631] | 477 [355-624] | 0.47 |
| **CRP on ICU admission, mg/dL** | 147 [86-235] | 150.4 [88-244] | 0.73 |
| **Mechanical Ventilation, n (%)** | 1128 (61.2) | 999 (57.1) | 0.20 |
| **ECMO, n (%)** | 75 (4.1) | 63 (3.6) | 0.65 |
| **Secondary Infection, n (%)** | 707 (49.5) | 664 (38.0) | 0.63 |
|  |  |  |  |
| **Hydroxychloroquine, n (%)** | 1441 (75.9) | 1308 (74.8) | 0.58 |
| **Lopinavir/Ritonavir, n (%)** | 19 (1.0) | 18 (1.0) | 0.99 |
| **Remdesivir, n (%)** | 19 (1.0) | 18 (1.0) | 0.99 |
| **Tocilizumab, n (%)** | 38 (2.0) | 36 (2.0) | 0.90 |
| **Macrolides, n (%)** | 267 (14.1) | 247 (14.1) | 0.90 |
| **Corticosteroids, n (%)** | 359 (21.6) | 332 (21.7) | 0.99 |
|  |  |  |  |
| **ICU length of stay, days** | 8 [4-17] | 9 [4-19] | 0.37 |
| **Hospital length of stay, days** | 17 [9-30] | 17 [10-31] | 0.08 |
|  |  |  |  |
| **General Hospital, n (%)** | 1204 (63.5) | 1086 (62.4) | 0.52 |
| **General Hospital with University Characteristics, n (%)** | 412 (21.7) | 381 (21.8) | 0.94 |
| **University Hospital, n (%)** | 280 (14.7) | 273 (15.6) | 0.47 |
|  |  |  |  |
| **Number of recognized ICU beds** | 22 [12-36] | 22 [13-36] | 0.07 |
| **Proportion between created and total ICU beds** | 0.38 [0.30-0.48] | 0.38 [0.30-0.48] | 0.69 |
| **ICU overflow, n (%)** | 851 (48.3) | 745 (46.1) | 0.21 |

ICU = intensive care unit; ECMO = extracorporeal membrane oxygenation; ACEIs = angiotensin converting enzyme inhibitors; ARBs = angiotensin II receptor blockers; RT-PCR = real-time polymerase chain reaction

**Supplemental eTable 2.** Characteristics of study population, according to the in-hospital mortality, including only patients treated with invasive mechanical ventilation. COVID-19 hospital clinical surveillance, Belgium, March 1^st^ – August 9^th^ 2020. Missing values are related to patients with available outcome data (n=999). Data are presented as median [IQRs] or count (%).

|  | **Survivors**  **(n=512)** | **Non-Survivors**  **(n=487)** | ***Missing*** | ***p value*** |
| --- | --- | --- | --- | --- |
| **Age, years** | 62 [54-71] | 70 [62-76] | - | <0.001 |
| *Age <=30, n (%)* | 8 (1.5) | 1 (0.2) | - | 0.04 |
| *Age 31-40, n (%)* | 18 (3.5) | 4 (0.8) | - | 0.004 |
| *Age 41-50, n (%)* | 66 (12.9) | 18 (3.7) | - | <0.001 |
| *Age 51-60, n (%)* | 134 (26.1) | 86 (17.6) | - | 0.002 |
| *Age 61-70, n (%)* | 150 (29.2) | 136 (45.5) | - | 0.68 |
| *Age 71-80, n (%)* | 121 (23.6) | 175 (35.9) | - | <0.001 |
| *Age >80, n (%)* | 15 (2.9) | 67 (13.7) | - | <0.001 |
| **Male Gender, n (%)** | 345 (67.3) | 338 (69.4) | - | 0.33 |
|  |  |  |  |  |
| **Cardiovascular Disease, n (%)** | 134 (26.1) | 181 (37.1) | - | <0.001 |
| **Arterial Hypertension, n (%)** | 229 (44.7) | 225 (46.2) | - | 0.69 |
| **Diabetes, n (%)** | 137 (26.7) | 132 (27.1) | - | 0.96 |
| **Obesity, n (%)** | 72 (14.0) | 53 (10.8) | 443 | 0.29 |
| **Chronic Pulmonary Disease, n (%)** | 56 (10.9) | 78 (16.0) | - | 0.02 |
| **Chronic Neurological Disease, n (%)** | 29 (5.6) | 24 (4.9) | - | 0.67 |
| **Chronic Cognitive Deficit, n (%)** | 14 (4.0) | 15 (3.0) | 116 | 0.85 |
| **Chronic Renal Disease, n (%)** | 39 (7.6) | 75 (15.4) | - | <0.001 |
| **Liver Cirrhosis, n (%)** | 15 (2.9) | 17 (3.4) | - | 0.72 |
| **Solid Cancer, n (%)** | 28 (5.4) | 26 (5.3) | - | 0.99 |
| **Hematological Cancer, n (%)** | 9 (1.7) | 11 (2.2) | 19 | 0.65 |
| **Chronic Immunosuppression, n (%)** | 12 (2.3) | 19 (3.9) | - | 0.21 |
| **Pregnancy/Post-partum, n (%)** | 3 (0.6) | 1 (0.2) | - | 0.63 |
| **No comorbidities, n (%)** | 143 (27.9) | 89 (18.2) | - | <0.001 |
| **Smoking, n (%)** | 21 (4.1) | 27 (5.5) | - | 0.17 |
| **ACEIs and/or ARBs, n (%)** | 156 (30.4) | 139 (28.5) | 77 | 0.56 |
|  |  |  |  |  |
| **Travel to at-risk region, n (%)** | 20 (3.9) | 15 (3.0) | - | 0.49 |
| **Health-care worker, n (%)** | 14 (2.7) | 6 (1.2) | - | 0.11 |
| **Nursing home resident, n (%)** | 18 (3.5) | 42 (8.6) | - | <0.001 |
|  |  |  |  |  |
| **Days from symptoms to hospital admission** | 6 [3-8] | 5 [2-7] | - | 0.001 |
| **Admitted for clinical reasons, n (%)** | 474 (92.5) | 433 (88.9) | - | 0.11 |
| **Admitted as at-risk patient, n (%)** | 74 (14.4) | 74 (15.1) | - | 0.76 |
| **Secondary Transfer, n (%)** | 29 (5.6) | 21 (4.3) | - | 0.37 |
| **Diagnosis by RT-PCR, n (%)** | 467 (91.2) | 438 (89.9) | - | 0.56 |
| **Diagnosis by chest CT-scan, n (%)** | 177 (34.5) | 156 (32.0) | 453 | 0.88 |
| **Diagnosis by rapid antigen method, n (%)** | 9 (1.7) | 14 (2.8) | 455 | 0.21 |
| **Body temperature on admission,** °**C** | 37.8 [37.0-38.6] | 37.7 [36.8-38.3] | - | 0.007 |
| **Fever, n (%)** | 397 (77.5) | 344 (70.6) | - | 0.02 |
| **Weakness, n (%)** | 222 (43.3) | 185 (37.9) | - | 0.096 |
| **Cough, n (%)** | 338 (66.0) | 271 (55.6) | - | <0.001 |
| **Throat Pain, n (%)** | 41 (8.0) | 18 (3.6) | - | 0.006 |
| **Runny nose, n (%)** | 28 (5.4) | 19 (3.9) | - | 0.002 |
| **Anosmia, n (%)** | 22 (4.3) | 10 (2.1) | 146 | 0.048 |
| **Breathless/Dyspnea, n (%)** | 345 (67.3) | 323 (66.3) | - | 0.77 |
| **Diarrhea, n (%)** | 91 (17.7) | 67 (13.7) | - | 0.10 |
| **Nausea/Vomit, n (%)** | 47 (9.1) | 35 (7.1) | - | 0.30 |
| **Headache, n (%)** | 71 (13.8) | 37 (7.6) | - | 0.002 |
| **Mental disorders, n (%)** | 23 (4.4) | 29 (5.9) | - | 0.37 |
| **Coma, n (%)** | 10 (1.9) | 17 (3.4) | - | 0.17 |
| **Convulsions, n (%)** | 1 (0.2) | 0 (0) | - | 0.99 |
| **Pharyngitis, n (%)** | 9 (1.7) | 11 (2.2) | - | 0.65 |
| **Conjunctivitis, n (%)** | 4 (0.8) | 4 (0.8) | - | 0.68 |
| **Asymptomatic, n (%)** | 6 (1.2) | 6 (1.2) | - | 0.99 |
| **No clinical signs, n (%)** | 26 (5.1) | 29 (5.9) | - | 0.63 |
| **Pneumonia at chest-X rays, n (%)** | 491 (95.8) | 464 (95.2) | 11 | 0.88 |
| **Day from hospital to ICU admission** | 1 [0-4] | 1 [0-3] | - | 0.43 |
| **Day from symptoms to ICU admission** | 8 [5-10] | 7 [3-10] | - | <0.001 |
| **PaO_2_ on ICU admission, mmHg** | 68 [54-82] | 67 [55-80] | 134 | 0.88 |
| **PaCO_2_ on ICU admission, mmHg** | 36 [31-42] | 37 [32-45] | 59 | 0.01 |
| **pH on ICU admission** | 7.47 [7.41-7.49] | 7.43 [7.34-7.48] | 247 | <0.001 |
| **Lactate on ICU admission, mmol/L** | 1.2 [0.9-1.8] | 1.4 [1.1-2.5] | 248 | <0.001 |
| **Creatinine on ICU admission, mg/dL** | 0.90 [0.70-1.21] | 1.33 [0.89-6.83] | 248 | <0.001 |
| **Lymphocytes on ICU admission, n/mm^3^** | 670 [219-1205] | 410 [14-800] | 311 | <0.001 |
| **LDH on ICU admission, IU/L** | 478 [371-613] | 546 [414-724] | 340 | <0.001 |
| **CRP on ICU admission, mg/dL** | 167 [101-249] | 187 [110-269] | 419 | 0.12 |
|  |  |  |  |  |
| **ECMO, n (%)** | 20 (3.9) | 40 (8.2) | 26 | 0.005 |
| **Secondary Infection, n (%)** | 227 (44.3) | 248 (50.9) | 131 | 0.29 |
|  |  |  |  |  |
| **Hydroxychloroquine, n (%)** | 441 (86.1) | 369 (75.7) | 4 | <0.001 |
| **Lopinavir/Ritonavir, n (%)** | 7 (1.3) | 8 (1.6) | 6 | 0.80 |
| **Remdesivir, n (%)** | 14 (2.6) | 3 (0.6) | 7 | 0.01 |
| **Tocilizumab, n (%)** | 15 (2.9) | 11 (2.2) | 8 | 0.55 |
| **Macrolides, n (%)** | 84 (16.4) | 64 (13.1) | 7 | 0.15 |
| **Corticosteroids, n (%)** | 125 (24.4) | 106 (21.7) | 134 | 0.48 |
|  |  |  |  |  |
| **ICU length of stay, days** | 20 [12-30] | 12 [7-21] | - | <0.001 |
| **Hospital length of stay, days** | 34 [22-50] | 14 [8-25] | - | <0.001 |
|  |  |  |  |  |
| **General Hospital, n (%)** | 314 (61.3) | 315 (64.6) | 5 | 0.31 |
| **General Hospital with University Characteristics, n (%)** | 100 (19.5) | 118 (24.2) | 5 | 0.008 |
| **University Hospital, n (%)** | 95 (18.5) | 52 (10.6) | 5 | <0.001 |
|  |  |  |  |  |
| **Number of recognized ICU beds** | 24 [14-39] | 22 [14-39] | 5 | 0.18 |
| **Ratio between created and total ICU beds** | 0.37 [0.26-0.44] | 0.38 [0.30-0.45] | 5 | <0.001 |
| **Ratio between available and occupied COVID-19 ICU beds** | 0.89 [0.65-1.31] | 1.01 [0.70-1.35] | 68 | 0.02 |
| **ICU overflow, n (%)** | 203 (41.7) | 225 (50.5) | 68 | 0.008 |

ICU = intensive care unit; ECMO = extracorporeal membrane oxygenation; ACEIs = angiotensin converting enzyme inhibitors; ARBs = angiotensin II receptor blockers; RT-PCR = real-time polymerase chain reaction

**Supplemental eTable 3.** Characteristics of study population, comparing the whole cohort to the one including only patients with available biological data on ICU admission (i.e. “selected patients”). COVID-19 hospital clinical surveillance, Belgium, March 1^st^ – August 9^th^ 2020. Data are presented as median [IQRs] or count (%).

|  | **All Patients (n=1903)** | **Selected Patients**  **(n=823)** | ***p value*** |
| --- | --- | --- | --- |
| **Age, years** | 65 [55-75] | 64 [55-73] | 0.31 |
| **Male Gender, n (%)** | 1287 (67.6) | 572 (69.5) | 0.47 |
|  |  |  |  |
| **Cardiovascular Disease, n (%)** | 602 (31.6) | 272 (33.0) | 0.49 |
| **Arterial Hypertension, n (%)** | 831 (43.6) | 378 (45.9) | 0.29 |
| **Diabetes, n (%)** | 484 (25.4) | 213 (25.8) | 0.84 |
| **Obesity, n (%)** | 225 (19.3) | 104 (12.6) | 0.28 |
| **Chronic Pulmonary Disease, n (%)** | 283 (14.8) | 112 (13.6) | 0.42 |
| **Chronic Neurological Disease, n (%)** | 102 (5.3) | 45 (5.4) | 0.98 |
| **Chronic Cognitive Deficit, n (%)** | 72 (4.1) | 41 (4.9) | 0.14 |
| **Chronic Renal Disease, n (%)** | 198 (10.4) | 101 (12.2) | 0.17 |
| **Liver Cirrhosis, n (%)** | 68 (3.5) | 32 (3.8) | 0.77 |
| **Solid Cancer, n (%)** | 113 (5.9) | 50 (6.0) | 0.96 |
| **Hematological Cancer, n (%)** | 40 (2.1) | 15 (1.8) | 0.77 |
| **Chronic Immunosuppression, n (%)** | 52 (2.7) | 31 (3.6) | 0.19 |
| **Pregnancy/Post-partum, n (%)** | 10 (0.5) | 6 (0.7) | 0.59 |
| **No comorbidities, n (%)** | 452 (23.7) | 187 (22.7) | 0.59 |
| **Smoking, n (%)** | 110 (10.5) | 42 (5.1) | 0.32 |
| **ACEIs and/or ARBs, n (%)** | 531 (27.9) | 258 (31.3) | 0.12 |
|  |  |  |  |
| **Travel to at-risk region, n (%)** | 71 (4.0) | 22 (2.6) | 0.39 |
| **Health-care worker, n (%)** | 64 (3.3) | 59 (7.1) | 0.61 |
| **Nursing home resident, n (%)** | 124 (6.5) | 61 (7.4) | 0.74 |
|  |  |  |  |
| **Days from symptoms to hospital admission** | 6 [3-8] | 6 [3-8] | 0.32 |
| **Admitted for clinical reasons, n (%)** | 1702 (89.9) | 750 (91.1) | 0.17 |
| **Admitted as at-risk patient, n (%)** | 227 (12.6) | 142 (17.2) | **<0.001** |
| **Diagnosis by RT-PCR, n (%)** | 1696 (89.1) | 746 (90.6) | 0.26 |
| **Diagnosis by chest CT-scan, n (%)** | 671 (58.2) | 274 (33.2) | 0.94 |
| **Diagnosis by rapid antigen method, n (%)** | 42 (3.6) | 19 (2.3) | 0.67 |
| **Mechanical Ventilation, n (%)** | 1128 (61.1) | 503 (61.1) | 0.58 |
| **ECMO, n (%)** | 75 (4.1) | 35 (4.2) | 0.86 |
| **Secondary Infection, n (%)** | 707 (49.5) | 400 (48.6) | 0.31 |
|  |  |  |  |
| **Hydroxychloroquine, n (%)** | 1441 (75.9) | 678 (82.3) | **<0.001** |
| **Lopinavir/Ritonavir, n (%)** | 19 (1.0) | 9 (1.0) | 0.84 |
| **Remdesivir, n (%)** | 19 (1.0) | 8 (0.9) | 0.99 |
| **Tolicizumab, n (%)** | 38 (2.0) | 20 (2.4) | 0.47 |
| **Macrolides, n (%)** | 267 (14.1) | 98 (11.9) | 0.14 |
| **Corticosteroids, n (%)** | 359 (21.6) | 143 (50.1) | 0.22 |
|  |  |  |  |
| **ICU length of stay, days** | 8 [4-17] | 9 [4-18] | 0.54 |
| **Hospital length of stay, days** | 17 [9-30] | 16 [9-28] | 0.24 |
| **Week confirmation date** | 14 [13-15] | 13 [13-15] | **0.04** |
|  |  |  |  |
| **General Hospital, n (%)** | 1204 (63.5) | 432 (52.4) | **<0.001** |
| **General Hospital University-affiliated, n (%)** | 412 (21.7) | 214 (26.0) | **0.02** |
| **University Hospital, n (%)** | 280 (14.7) | 177 (21.5) | **<0.001** |
|  |  |  |  |
| **Public Hospital, n (%)** | 474 (25.0) | 169 (20.5) | 0.14 |
| **Private Hospital, n (%)** | 1422 (75.0) | 654 (79.5) | .0.14 |
|  |  |  |  |
| **Number of recognized ICU beds** | 22 [12-36] | 24 [14-39] | **<0.001** |
| **Ratio between created and total ICU beds** | 0.38 [0.30-0.48] | 0.42 [0.31-0.49] | **0.01** |
| **Ratio between available and occupied COVID-19 ICU beds** | 0.98 [0.68-1.39] | 1.00 [0.69-1.38] | 0.73 |
|  |  |  |  |
| **Hospital Mortality, n (%)** | 632 (36.1) | 467 (61.2%) | 0.33 |
