# Supplementary material for: The role of organizational characteristics on the outcome of COVID-19 patients admitted to the ICU in Belgium
Source: Lancet Reg Health Eur. 2020 Dec 23;2:100019. doi: 10.1016/j.lanepe.2020.100019 (PMC7757349; doi:10.1016/j.lanepe.2020.100019)
Supplement: Supplementary file 2 [file mmc2.docx]

**Group Information**:

1. The Board members of the Belgian Society of Intensive Care Medicine:
   1. P Biston
   2. V Collin
   3. D Ledoux
   4. G Hermans
   5. D Nam Nguyen
   6. K De Decker
   7. P Jorens
   8. P Depuydt
   9. T Sottiaux
2. The collaborative group on COVID-19 Hospital surveillance in Belgium include:
   1. SA Aouachria
   2. K Bafort
   3. L Belkhir
   4. N Bossuyt
   5. V Colombie
   6. N Dauby
   7. P De Munter
   8. J Deblonde
   9. D Delmarcelle
   10. M Delvallee
   11. R Demeester
   12. T Dugernier
   13. X Holemans
   14. B Kerzmann,
   15. PY Machurot
   16. P Minette
   17. JM Minon
   18. S Mokrane
   19. C Nachtergal
   20. S Noirhomme
   21. D Piérard
   22. C Rossi
   23. C Schirvel
   24. E Sermijn
   25. F Staelens
   26. F Triest
   27. J Van Praet
   28. A Vanhoenacker
   29. R Verstraete
   30. E Willems
   31. C Wyndham-Thomas
