# Supplementary material for: The role of organizational characteristics on the outcome of COVID-19 patients admitted to the ICU in Belgium
Source: Lancet Reg Health Eur. 2020 Dec 23;2:100019. doi: 10.1016/j.lanepe.2020.100019 (PMC7757349; doi:10.1016/j.lanepe.2020.100019)
Supplement: Supplementary file 3 [file mmc3.docx]

*The following translations in French, Italian, Português, Spanish, Netherlands, German were submitted by the authors and we reproduce them as supplied. They have not been peer reviewed. Our editorial processes have only been applied to the original abstract in English, which should serve as reference for this manuscript.*

**Le rôle des caractéristiques organisationnelles sur le devenir des patients COVID-19 admis à l'USI en Belgique (FRENCH)**

**Résumé**

*Contexte:* Plusieurs études ont examiné les prédicteurs de la mortalité hospitalière chez les patients atteints de COVID-19 qui doivent être admis en Réanimation. Cependant, aucune donnée n’est disponible sur le rôle des problèmes organisationnels sur l’évolution des patients dans ce contexte. Le but de cette étude était donc d'évaluer le rôle des facteurs organisationnels sur le devenir des patients COVID-19 graves admis en Réanimation en Belgique.

*Méthodes:* Nous avons réalisé une analyse rétrospective en analysant la mortalité hospitalière chez les patients COVID-19 admis en Réanimation en Belgique via la base de données nationale de surveillance. Les non-survivants à la sortie de l'hôpital ont été comparés aux survivants en utilisant une analyse de régression logistique à effets mixtes multivariés. Des analyses spécifiques incluant uniquement des patients avec ventilation invasive ont été réalisées. Pour évaluer les facteurs organisationnels, les données ont été fusionnées avec des informations administratives sur le type d'hôpital, le nombre de lits de Réanimation reconnus, le nombre de lits supplémentaires spécifiquement créés pour les patients COVID-19 et le facteur «surcharge USI» (c'est-à-dire, le rapport entre le nombre de lits occupés en Réanimation par les patients COVID-19 confirmés ou suspectés divisé par le nombre de lits de Réanimation reconnus réservés aux patients COVID-19; la surcharge était définie par un rapport ≥ 1.0).

*Résultats*: Sur un total de 13612 patients hospitalisés atteints de la COVID-19 avec des données d'admission et de sortie enregistrées au cours de la période de surveillance (du 1 Mars au 9 Août 2020), 1903 (14.0%) ont dû être hospitalisés en Réanimation, dont 1747 avaient des données de statut vital disponibles. Les non-survivants (n = 632, 36.1%) étaient plus âgés et avaient plus fréquemment des comorbidités que les survivants. Dans l'analyse multivariée, la surcharge USI, associée à un âge plus avancé, à la présence de comorbidités, à un délai plus court entre l'apparition des symptômes et l'hospitalisation, à l'absence de traitement à l'hydroxychloroquine et à l'utilisation de la ventilation mécanique invasive et de l'ECMO, était indépendamment associés à une augmentation de la mortalité hospitalière. Des résultats similaires ont été trouvés dans le sous-groupe de patients sous ventilation invasive. En plus, la proportion de lits supplémentaires spécialement créés pour la COVID-19 par rapport au nombre total de lits de Réanimation précédemment existant était associée à une augmentation de la mortalité hospitalière chez les patients sous ventilation invasive. Le modèle a également indiqué une différence significative de mortalité entre les hôpitaux, non expliquée par les données disponibles et les caractéristiques des hôpitaux.

*Interpretation*: Des facteurs organisationnels, comme la surcharge en Réanimation ou la création de lits supplémentaires spécifiques au COVID-19, étaient associés à une surmortalité chez les patients admis en Réanimation.

*Fonds*: Aucun.

**Il ruolo delle caratteristiche organizzative sull'esito dei pazienti COVID-19 ricoverati in Terapia Intensiva in Belgio (ITALIAN)**

**Riassunto**

*Introduzione:* Vari studi hanno esaminato i predittori di mortalità intraospedaliera per i pazienti COVID-19 che necessitano di essere ricoverati in unità di Terapia Intensiva (TI). Tuttavia, in questo contesto non sono disponibili dati sul ruolo dei fattori organizzativi sulla mortalità dei pazienti. Lo scopo di questo studio era quindi quello di valutare il ruolo delle caratteristiche organizzative sull'esito dei pazienti con COVID-19 in condizioni critiche ammessi in TI in Belgio.

*Metodi*: Abbiamo condotto un'analisi retrospettiva della mortalità intraospedaliera nei pazienti COVID-19 ammessi in TI in Belgio tramite il database di sorveglianza nazionale. I deceduti alla dimissione dall'ospedale sono stati confrontati con i sopravvissuti utilizzando un'analisi di regressione logistica multivariabile a effetti misti. Delle analisi specifiche sono state eseguite includendo solo i pazienti con ventilazione invasiva. Per valutare i fattori organizzativi, i dati sono stati uniti alle informazioni amministrative sul tipo di ospedale, il numero di base di letti in TI riconosciuti, il numero di letti supplementari in TI creati appositamente per i pazienti COVID-19 e il "sovraccarico in TI" (cioè il rapporto tra il numero di letti in TI occupati da pazienti COVID-19 confermati o sospetti diviso per il numero di letti in TI riconosciuti riservati ai pazienti con COVID-19; il sovraccarico era presente se il rapporto é ≥ 1.0).

*Risultati*: Su un totale di 13612 pazienti COVID-19 ricoverati con moduli di ammissione e dimissione registrati nel periodo di sorveglianza (dal 1 marzo al 9 agosto 2020), 1903 (14.0%) hanno richiesto il ricovero in TI, di cui 1747 avevano dati disponibili sulla mortalità. I deceduti (n = 632, 36.1%) erano più anziani e avevano più frequentemente varie malattie concomitanti rispetto ai sopravvissuti. Nell'analisi multivariata, il sovraccarico in TI, insieme all'età avanzata, presenza di comorbidità, tempo più breve tra l'insorgenza dei sintomi e il ricovero ospedaliero, l'assenza di terapia con idrossiclorochina e l'uso della ventilazione meccanica invasiva e dell'ECMO, era associato indipendentemente con un aumento della mortalità ospedaliera. Risultati simili sono stati osservati nel sottogruppo di pazienti ventilati invasivamente. Inoltre, la proporzione di letti supplementari creati per il COVID-19 in TI rispetto al numero totale di letti in TI precedentemente esistente era associata a un aumento della mortalità ospedaliera tra i pazienti con ventilazione invasiva. Il modello ha anche indicato una differenza significativa nella mortalità ospedaliera tra ospedali, non spiegata dai dati disponibili e dalle caratteristiche ospedaliere.

*Conclusioni*: È stato riscontrato che dei fattori organizzativi, come il sovraccarico in TI o la creazione di letti supplementari di TI per COVID-19, hanno un impatto negativo sul divenire dei pazienti COVID-19.

*Fondi*: Nessuno.

**O papel das características organizacionais e estruturais no desfecho de pacientes com COVID-19 internados em UTIs na Bélgica (PORTUGUÊS)**

**Resumo**

*Introdução*: Diversos estudos investigaram os preditores de mortalidade intra-hospitalar em pacientes com COVID-19 internados em Unidades de Terapia Intensiva (UTIs). No entanto, não existem dados disponíveis relativos ao impacto de questões estruturais e organizacionais no desfecho destes pacientes. Portanto, o objetivo deste estudo foi avaliar o papel destas questões organizacionais e estruturais no desfecho de pacientes críticos com COVID-19 admitidos em UTIs na Bélgica.

*Métodos*: Nós realizamos uma análise retrospectiva da mortalidade intra-hospitalar de pacientes com COVID-19 internados em UTIs belgsa através de dados obtidos pelo sistema nacional de vigilância. Pacientes que morreram durante a internação hospitalar foram comparados a sobreviventes através de um modelo de regressão logística multivariável com efeitos mistos. Análises específicas incluindo apenas pacientes com ventilação invasiva também foram realizadas. Para avaliar a capacidade de resposta ao surto, nós combinamos os dados clínicos com informações administrativas sobre o tipo de hospital, o número de base de leitos de UTI recohecidos, o número de leitos de UTI suplementares criados especificamente para os cuidados de pacientes COVID-19 e a "sobrecarga de UTI" (proporção variável ao longo do tempo entre o número de leitos de UTI ocupados por pacientes confirmados e suspeitos de COVID-19 dividido pelo número de leitos de UTI reconhecidos reservados para pacientes com COVID-19; quando essa proporção é ≥ 1,0 consideramos que a capacidade de ocupação da UTI foi ultrapassada e confirmamos a sobrecarga).

*Resultados:* De um total de 13.612 pacientes COVID-19 hospitalizados com formulários de admissão e alta registrados no período de vigilância (1 de março a 9 de agosto de 2020), 1.903 (14,0%) necessitaram de internação em UTI, dos quais 1.747 tinham dados de desfecho disponíveis. Os não sobreviventes (n = 632, 36,1%) eram mais velhos e tinham mais comorbidades do que os sobreviventes. Na análise multivariável, a sobrecarga da capacidade de ocupação da UTI, juntamente com idade avançada, presença de comorbidades, menor duração do tempo entre o início dos sintomas e a admissão hospitalar, ausência de tratamento com hidroxicloroquina e uso de ventilação mecânica invasiva e de ECMO, foram independentemente associados a um aumento da mortalidade intra-hospitalar . Resultados semelhantes foram encontrados no subgrupo de pacientes ventilados invasivamente. Além disso, nesse subgrupo de pacientes, a proporção de leitos de UTI suplementares criados especificamente para internação de pacientes COVID-19 em relação ao número total de leitos da UTI previamente existente foi associada a um aumento da mortalidade. O modelo também indicou uma diferença significativa entre hospitais em relação a mortalidade intra-hospitalar, não explicada pelas características dos pacientes e dos hospitais.

*Interpretação:* A capacidade estrutural e organizacional de se adequar ao aumento do fluxo de pacients refletida pela sobrecarga da capacidade de ocupação da UTI e pela criação de leitos de UTI suplementares específicos COVID-19 teve um impacto negativo no desfecho dos pacientes internados em UTIs belgas.

*Financiamento:* Nenhuma financiamento externo utiliado para a realização deste estudo.

**El rol de las características organizativas en el desenlace final de los pacientes con COVID-19 ingresados ​​en la UCI de Bélgica (SPANISH)**

**Resumen**

*Antecedentes*: Varios estudios han investigado los predictores de mortalidad intrahospitalaria para pacientes con COVID-19 que necesitan ser ingresados ​​en la Unidad de Cuidados Intensivos (UCI). Sin embargo, en este contexto no se dispone de datos sobre el rol de las cuestiones organizativas en el desenlace final de los pacientes. Por lo tanto, el objetivo de este estudio fue evaluar el rol de la organización de la capacidad de reacción en el desenlace final de los pacientes con COVID-19 en estado crítico ingresados ​​en UCI en Bélgica.

*Métodos:* Realizamos un análisis retrospectivo de la mortalidad hospitalaria en pacientes con COVID-19 en la UCI belga a través de la base de datos de vigilancia nacional. Los que no sobrevivieron al alta hospitalaria se compararon con los que sobrevivieron mediante un análisis de regresión logística multivariable de efectos mixtos. Se realizaron análisis específicos que incluyeron solo a pacientes con ventilación invasiva. Para evaluar la capacidad de reacción, los datos se combinaron con la información administrativa sobre el tipo de hospital, el número inicial de camas de UCI reconocidas, el número de camas suplementarias creadas específicamente para la atención de COVID-19 en la UCI y el "desbordamiento de la UCI" (es decir, una relación variable en el tiempo entre el número de camas de UCI ocupadas por pacientes confirmados y sospechosos de COVID-19 dividido por el número de camas de UCI reconocidas reservadas para pacientes con COVID-19; el desbordamiento de UCI se presenta cuando esta relación es ≥ 1.0).

*Resultados:* Sobre un total de 13.612 pacientes COVID-19 hospitalizados con formularios de admisión y alta registrados en el período de vigilancia (del 1 de marzo al 9 de agosto de 2020), 1903 (14.0%) requirieron ingreso en la UCI, de los cuales 1747 tenían datos de desenlaces finales disponibles. Los no sobrevivientes (n = 632, 36.1%) eran mayores y tenían varias enfermedades comórbidas con más frecuencia que los sobrevivientes. En el análisis multivariable, el desbordamiento de la UCI, junto con la edad avanzada, la presencia de comorbilidades, el menor retraso entre el inicio de los síntomas y el ingreso hospitalario, la ausencia de tratamiento con hidroxicloroquina y el uso de ventilación mecánica invasiva y de ECMO, se asoció de forma independiente con una mayor mortalidad hospitalaria. . Se encontraron resultados similares entre el subgrupo de pacientes con ventilación invasiva. Además, la proporción de camas suplementarias creadas específicamente para la atención de COVID-19 en la UCI con respecto al número total de camas de la UCI previamente existentes se asoció con un aumento de la mortalidad hospitalaria entre los pacientes con ventilación invasiva. El modelo también indicó una diferencia significativa entre hospitales en la mortalidad intrahospitalaria, no explicada por los pacientes disponibles ni las características hospitalarias.

*Interpretación:* Se encontró que la organización de la capacidad de reacción como se refleja en el desbordamiento de la UCI o la creación de camas de UCI suplementarias específicas de COVID-19 impactan negativamente en los desenlaces finales de los pacientes de la UCI.

*Financiamiento:* Ninguna fuente de financiamiento estuvo disponible para este estudio.

**De rol van organisatorische kenmerken op de uitkomst van COVID-19-patiënten opgenomen op een intensieve zorg afdeling in België (NEDERLANDS)**

**Abstract**

*Achtergrond*: Verschillende studies hebben reeds de voorspellende factoren voor ziekenhuissterfte van COVID-19 patiënten opgenomen op een intensieve zorg (IZ) afdeling onderzocht. Er zijn echter geen gegevens beschikbaar over de rol van organisatorische aspecten op de uitkomst van patiënten in deze setting. Het doel van deze studie was om de rol van de organisatie van de “Surge Capacity” te evalueren op de uitkomst van ernstig zieke COVID-19 patiënten die op de IZ afdelingen in België zijn opgenomen.

*Methoden*: We voerden een retrospectieve analyse uit van de ziekenhuissterfte bij Belgische COVID-19 patiënten opgenomen op een IZ afdeling op basis van nationale ziekenhuissurveillancegegevens. Niet-overlevenden bij ziekenhuisontslag werden vergeleken met overlevenden met behulp van multivariabele mixed effects logistieke regressieanalyse. Er werd eveneens een subgroepanalyse uitgevoerd voor de patiënten behandeld met invasieve ventilatie. Om de invloed van ziekenhuiscapaciteit (Surge Capacity) op mortaliteit te beoordelen werden gegevens samengevoegd met administratieve informatie over het type ziekenhuis, het aantal erkende IZ-bedden, het aantal aanvullende bedden dat specifiek voor COVID-19 intensieve zorg werd gecreëerd, en de IZ “overflow” (d.w.z. een tijdsafhankelijke ratio tussen het aantal bezette IZ-bedden door bevestigde en verdachte COVID-19-patiënten gedeeld door het aantal erkende IZ-bedden dat is voorbehouden voor COVID-19-patiënten; IZ “overflow” aanwezig wanneer deze ratio ≥ 1,0 is).

*Resultaten*: Op een totaal van 13.612 gehospitaliseerde COVID-19 patiënten met opname- en ontslagformulieren geregistreerd in de surveillanceperiode (1 maart tot en met 9 augustus 2020), werden 1.903 (14,0%) patiënten opgenomen op een IZ afdeling, waarvan voor 1.747 patiënten gegevens over de uitkomst beschikbaar waren. Niet-overlevenden (n=632, 36,1%) waren ouder en hadden vaker verschillende comorbide ziekten dan overlevenden. In de multivariabele analyse werd de IZ “overflow”, samen met de oudere leeftijd, de aanwezigheid van comorbiditeiten, de kortere tijd tussen het optreden van de symptomen en de opname in het ziekenhuis, de afwezigheid van hydroxychloroquine therapie, en het gebruik van invasieve mechanische ventilatie en van ECMO, onafhankelijk van elkaar in verband gebracht met een verhoogde sterfte in het ziekenhuis. Vergelijkbare resultaten werden gevonden bij de subgroep van invasief beademde patiënten. Daarnaast werd het aandeel van aanvullende bedden specifiek voor de COVID-19 intensieve zorg ten opzichte van het aantal erkende IZ-bedden geassocieerd met een verhoogde ziekenhuissterfte onder deze invasief beademde patiënten. Het model gaf ook een significant verschil in ziekenhuissterfte aan tussen de verschillende ziekenhuizen, dat niet verklaard kon worden aan de hand van de patiëntenprofielen of de organisatorische kenmerken.

*Interpretatie*: Organisatie van de “Surge Capacity” die resulteert in IZ “overflow” of het creëren van aanvullende IZ-bedden bovenop de erkende bedden specifiek voor de COVID-19 intesieve zorg bleek een negatief effect te hebben op de uitkomst van de COVID-19 patiënten die op de IZ-afdelingen in België werden opgenomen.

*Funding*: Geen.

**Die Rolle organisatorischer Charakteristiken für den medizinischen Ausgang von COVID-19-Patienten, die auf der Intensivstation in Belgien aufgenommen wurden (GERMAN)**

**Hintergrund:** In mehreren Studien wurden Faktoren untersucht, die es möglich machen die Krankenhausmortalität von COVID-19-Patienten, die auf der Intensivstation (ICU) aufgenommen werden müssen, vorauszusagen. In dieser Situation sind jedoch meist keine Daten zum Einfluss von organisatorischen Problemen auf den Behandlungsausgang der Patienten verfügbar. Ziel dieser Studie war es daher, die Rolle der Organisation im Krankenhause während der Phase in der die Schwallkapazität erreicht ist für den Behandlungsausgang kritisch kranker COVID-19-Patienten, die auf Intensivstationen in Belgien aufgenommen wurden, zu bewerten.

**Methoden:** Wir führten eine retrospektive Analyse der Krankenhausmortalität von belgischen COVID-19-Patienten auf der Intensivstation an Hand von Daten der nationalen Überwachungsdatenbank durch. Nichtüberlebende bei Entlassung aus dem Krankenhaus wurden mit Überlebenden verglichen, wobei eine multivariable logistische Regressionsanalyse mit gemischten Effekten verwendet wurde. Spezifische Analysen von Patienten mit invasiver Beatmung wurden durchgeführt. Zur Beurteilung des Erreichens der Schwallkapazität wurden Verwaltungsdaten mit Informationen über die Art des Krankenhauses, die Basisanzahl der anerkannten Intensivbetten, die Anzahl der speziell für die COVID-19-Intensivpflege erstellten Zusatzbetten und den „Intensivüberlauf“ (dh. das zeitlich variierende Verhältsnis zwischen der Anzahl der belegten Intensivbetten durch bestätigte und vermutete COVID-19-Patienten geteilt durch die Anzahl der anerkannten Intensivbetten, die für COVID-19-Patienten reserviert sind; Überlauf der Intensivstation vorhanden, wenn dieses Verhältnis ≥ 1,0 ist).

**Ergebnisse:** Von insgesamt 13.612 im Krankenhaus befindliche COVID-19-Patienten mit Aufnahme- und Entlassungsformularen, die im Überwachungszeitraum (1. März bis 9. August 2020) registriert waren, erforderten 1903 (14,0%) die Aufnahme auf die Intensivstation, darunter 1747 mit verfügbare Daten über den Behandlungsausgang. Nichtüberlebende (n = 632, 36,1%) waren älter und hatten häufiger verschiedene komorbide Erkrankungen als Überlebende. In der multivariablen Analyse war der Überlauf der Intensivstation zusammen mit einem höheren Alter, dem Vorhandensein von Komorbiditäten, der kürzeren Verzögerung zwischen dem Auftreten der Symptome und der Krankenhauseinweisung, dem Fehlen einer Hydroxychloroquin-Therapie und der Verwendung einer invasiven mechanischen Beatmung sowie von ECMO, unabhängig voneinander mit einer erhöhten Krankenhausmortalität verbunden. Ähnliche Ergebnisse wurden bei der Teilanalyse der invasiv beatmeten Patienten gefunden. Darüber hinaus war der Anteil von speziell für die COVID-19-Intensivpflege geschaffenen Zusatzbetten zu der Gesamtzahl zuvor vorhandener Intensivbetten mit einer erhöhten Krankenhausmortalität bei invasiv beatmeten Patienten verbunden. Das Modell zeigte auch einen signifikanten Unterschied zwischen den Krankenhäusern für die Krankenhausmortalität auf, der nicht durch die Merkmale der Patienten oder Krankenhäuser erklärt wurde.

**Interpretation:** Die Organisation der Schwallkapazität, die sich in einem Überlauf der Intensivstation oder der Schaffung von COVID-19-spezifischen zusätzlichen Intensivbetten widerspiegelt, wirkt sich negative auf den Behandlungsausgang von Patienten auf der Intensivstation aus.

**Finanzierung:** Für diese Studie war keine Finanzierungsquelle verfügbar.
